# Supplementary material for: Spatially-resolved fluorescence-detected two-dimensional electronic spectroscopy probes varying excitonic structure in photosynthetic bacteria
Source: Nat Commun. 2018 Oct 11;9:4219. doi: 10.1038/s41467-018-06619-x (PMC6181999; doi:10.1038/s41467-018-06619-x)
Supplement: Supplementary file 1 — Supporting Information [file 41467_2018_6619_MOESM1_ESM.pdf]

Supplementary Information for "Fluorescence-detected Two-dimensional Electronic Spectroscopy Spatially Resolves Varying Electronic Structure in Photosynthetic Bacteria"

Vivek Tiwari[1], Yassel Acosta Matutes[2], Alastair T. Gardiner[3], Thomas L. C. Jansen[4], Richard J. Cogdell[3] and Jennifer P. Ogilvie[1]

[1]Department of Physics, University of Michigan, Ann Arbor, MI, 48109

[2]Applied Physics Program, University of Michigan, Ann Arbor, MI, 48109

[3]Institute for Molecular Biology, University of Glasgow, Glasgow G12 8TA, United Kingdom

[4]University of Groningen, Zernike Institute for Advanced Materials, Groningen, the Netherlands

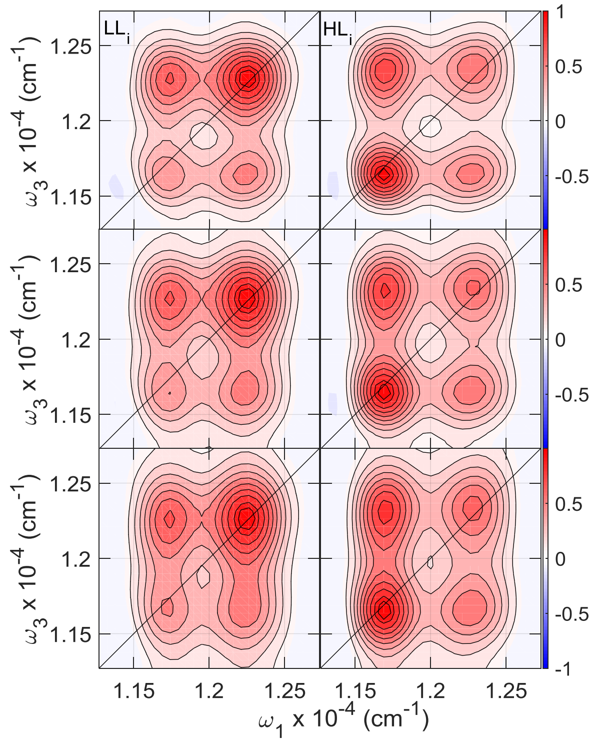

Supplementary Figure 1. Distortion of SF-2DES spectra due to photobleaching. **(Top)** "Ideal" SF-2DES spectra for the case when the sample is circulated and no drop in FWM signal or OD was observed after the experiment. These ideal experimentally measured spectra are marked as LL_i and HL_i, respectively. **(Middle)** Distortion in the above LL_i and HL_i spectra, when a constant exponential photobleaching rate is assumed in the experiment such that the total signal drops by 60% in the 45 seconds of data collection time. To simulate this effect, the experimentally collected consecutive time indices in the ideal data were multiplied by an exponentially decaying factor exp [-(*index number* x β)] , where β is a constant bleaching rate. For $\beta = 2.56 \times 10^{-3}$, the drop in the signal for 361 time indices collected in the experiment (19 steps from 0 to 90 fs in steps of 5 fs along each t_1 and t_3 time axes) was ~ 60%. Vertical distortions in the spectra are apparent, as is the rise in the pedestal in the middle of the four peaks. Vertical distortions along ω_3 axis are present because in the experiment t_1 delay is scanned for each successive value of t_3. Thus, a t_3 slice (at t_1 = 0 fs) is most affected by photobleaching, and a t_1 slice (at t_3 = 0 fs) is the least affected. The peaks become broader due to a faster decay in the time-domain, and begin to merge together. However, the diagonal and cross-peaks, which indicate exciton transition dipole strengths and Coulomb couplings, are still distinct. **(Bottom)** Distortion in the LL_i and HL_i spectra when the total signal drops by 80% in the 45 seconds of data collection time. For $\beta = 4.46 \times 10^{-3}$, the drop in the signal for 361 time indices collected in the experiment (19 steps from 0 to 90 fs in steps of 5 fs along each t_1 and t_3 time axes) was ~ 80%. Vertical distortions along ω_3 axis are increased compared to the middle panel, and the 2D peaks become further broadened. As noted above, in all cases, the photobleaching associated distortions are vertical in nature, and affect the relative cross-

peak amplitudes by 5% or lesser due to the peaks merging together along the detection axis. Contours are drawn at 10-90 % in steps of 10%, with additional contours at 95% and 100% to highlight small differences in maxima. The frequency ω corresponding to the axes on the SF-2DES plots corresponds to $\omega = |\omega'|/2\pi c$, where ω' is in rad/fs. All measurements were conducted at 300 K.

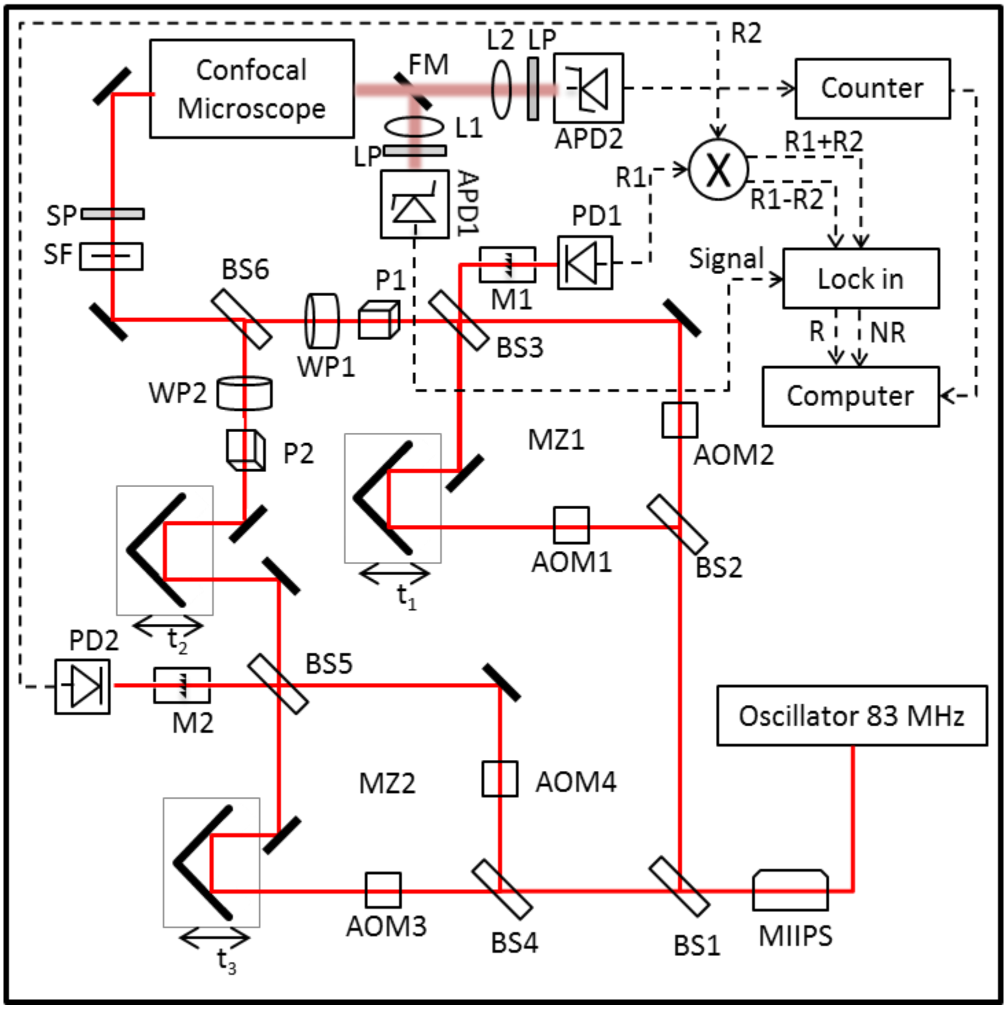

Supplementary Figure 2. Experimental layout of the spatially-resolved SF-2DES spectrometer.

Supplementary Note 1 – Details of Experimental Setup

The experimental layout is based on the original design by Marcus and co-workers[1]. The output from a commercial broadband 83 MHz Ti:Sapphire oscillator (Venteon One) was sent into a SLM based pulse shaper (MIIPS 640P, Biophotonic Solutions) for dispersion pre-compensation. Laser pulses from the pulse shaper of ~20 fs FWHM pulse duration are split by a 50:50 beamsplitter (Newport, 10B20BS.2), and each portion is routed into two Mach-Zehnder (MZ) interferometers. Within each of the two MZs, the pulse is further split using 50:50 beamsplitters (BS), and each arm is tagged with a unique phase which is modulated at radio frequency using an acousto-optic modulator (AOM, Isomet M1142-SF80L). The phase modulation frequencies for the four arms are denoted as Ω_{1-4}, such that when the split pulses are recombined at BS3 and BS5, within MZ1 and

MZ2 respectively, the pulse amplitude modulates at the difference frequencies of the AOMs, that is, Ω_{12} and Ω_{34} for MZ1 and MZ2, respectively. One output port from BS3 and BS5 is routed to a monochromator (Optometrics, MC1-04) to generate the reference frequencies, ω_1^R and ω_2^R for MZ1 and MZ2 respectively, which modulate at Ω_{12} and Ω_{34} difference frequencies, respectively. The references for all the SF-2DES experiments were set at 826 nm using a high-resolution spectrometer (Ocean Optics HR4000, 0.1 nm resolution). The FWHM of the reference spectra after the monochromators was 0.5 nm. Beams from the other two output ports (one from each of the two MZs) are each passed through a polarizer P (ThorLabs, GTH10M-B) and waveplate WP (ThorLabs, AHWP05M-980) combination, and are recombined at BS6. The train of 4 phase-modulated pulses generated by the two MZs is then routed through a spatial-filter with two 5 cm lenses, and a 25 μm pinhole in order to generate a TEM_{00} Gaussian spatially-filtered beam mode. Spatial filtering also helps to minimize any residual spatial chirp. The polarizations of the beams after the spatial filter is set to S to greater than 1300:1 extinction ratios for both MZs.

The resulting beam from the above SF-2DES Setup (Fig. 1, main text) passes through a OD4 875 SP filter (Edmund Optics), and is routed into a confocal microscope (Olympus 1X51). The microscope is set up in the epi-detection geometry. A 875 nm dichroic mirror (Semrock) inside the microscope transmits the pulse train towards the water objective (Olympus PlanApo 60x, NA1.2). The sample is drop-dried under Nitrogen on a 0.17 mm coverslip, which is sealed on a glass slide using double sided sticky tape followed by sealing the outside surface with Scotch tape. The objective collar is set to 0.17. The immobilized sample is mounted on an XY scanning piezo stage (Piezo Instruments, P-612.2SL). The fluorescence collected by the water objective is reflected by the dichroic mirror, spatially filtered through a 200 μm pinhole followed by optical filtering through an OD 4 875LP filter. Due to the large detection pinhole, the effect of the detection pinhole on the confocal point spread function will be negligible. For linear fluorescence imaging experiments, the filtered fluorescence is focused onto a single photon counting APD (APD1 operated in Geiger mode, Excelitas, SPCM-AQRH-16) using a 5 cm lens (ThorLabs LA1131-B). The photon counts registered by APD1 are counted using a counting circuit (National instruments, PCI 6110) for each XY position of the computer controlled piezo controller.

For SF-2DES measurements on the imaged sample, or for experiments in which the sample is circulated, the filtered fluorescence is routed by a flip mirror (FM) through a 5 cm lens

(ThorLabs LA1131-B) which focuses it on another APD operating in linear mode (APD2, Hamamatsu C12703-01). For experiments where the sample is circulated, the sample cuvette (Starna, 48-Q-0.2) has a 200 μm pathlength, and an air objective (Olympus LUCPlanFLN 40x, NA0.6) is used for the measurements. The sample was circulated through the cuvette using a peristaltic pump (Masterflex Model 07516-00) at flow rates of ~190 ml/min. For linear fluorescence imaging, the total incident power on the sample is ~0.125 μW/pulse, corresponding to a fluence of 0.26 μJ/cm^2/pulse. The binning time at each piezo location was 5 ms. For SF-2DES experiments on immobilized and circulating samples, the spectra were collected with a total power of 7.5uW/pulse, corresponding to a total fluence of 12-16 μJ/cm^2/pulse. At 83 MHz repetition rate, the *total* pulse energies for these fluences is ~ 0.09 pJ/pulse, such that the excitation probability at the center of the pulse is less than 0.1%. Low excitation probability is chosen so as to minimize multiple excitations on the molecule by the same pulse.

The references generated from MZs 1 and 2, which modulate at Ω_{12} and Ω_{34} AOM difference frequencies, respectively, are used for phase-sensitive lock-in detection, while the second port of MZ1 and MZ2 is recombined at BS6. The time delays between the all collinear train of 4 pulses after BS2 are controlled by translational stages (Newport M-VP25XL for t_1 and t_3 time delays, and ILS150 for t_2 time delay) within each arm of the MZs, resulting in an all-collinear train of 4 pulses separated by time delays t_1 between the first two pulses, t_3 between pulses 3 and 4, and t_2 between the 2nd and 3rd pulses in the sequence. t_1 and t_3 delays are scanned from 0 to 90 fs in steps of 5 fs. The t_2 delay is scannable from 0 to 800 ps, but is fixed at t_2 = 0 fs for the experiment.

The four-wave mixing signal generated by the sample oscillates at the difference frequencies $(\Omega_3 - \Omega_4) \pm (\Omega_2 - \Omega_1)$, where the (positive) negative sign corresponds to (non-rephasing) rephasing 2D signals. The oscillating signal is demodulated using a lock-in amplifier (Zurich Instruments, HF2LI). Physical undersampling of the oscillating signal, as well as phase-sensitive detection is achieved by signal detection relative to a reference frequency generated by passing MZ 1-2 outputs through monochromators 1-2. The reference signals from monochromators 1 and 2 modulate at Ω_{12} and Ω_{34} difference frequencies, respectively. The references are detected by amplified photodiodes PD1,2 (ThorLabs PDA36A) and mixed in a 24 bit digital signal processor (Analog Devices, ADAU1761) to generate the reference signals modulating at frequencies

$\Omega_{12} \pm \Omega_{34}$. These reference signals are connected to the lock-in amplifier reference channels corresponding to rephasing and non-rephasing signals. For the experiments, the AOM frequencies are set at Ω_1 = 80.111 MHz, Ω_2 = 80.101 MHz, Ω_3 = 80.029 MHz and Ω_4 = 80.0 MHz through a common clock (Novatech DDS Model 409B), such that the resulting signals oscillate at 19 kHz (rephasing) and 39 kHz (non-rephasing).

The 2D spectra obtained from desired XY locations on the fluorescence image were fit as a linear combination of the basis 2D spectra in Fig. 2. Two of the three coefficients of the linear combination were constrained to lie between 0 and 1, and the sum of the coefficients was constrained to 1. These coefficients correspond to the HL and LL contributions, and the third coefficient was an offset which was constant across a given 2D data, but was free to float across different data sets. In order to solve the constrained linear least-squares fitting, the MATLAB routine *lsqlin* was used with interior-point convex optimization algorithm.

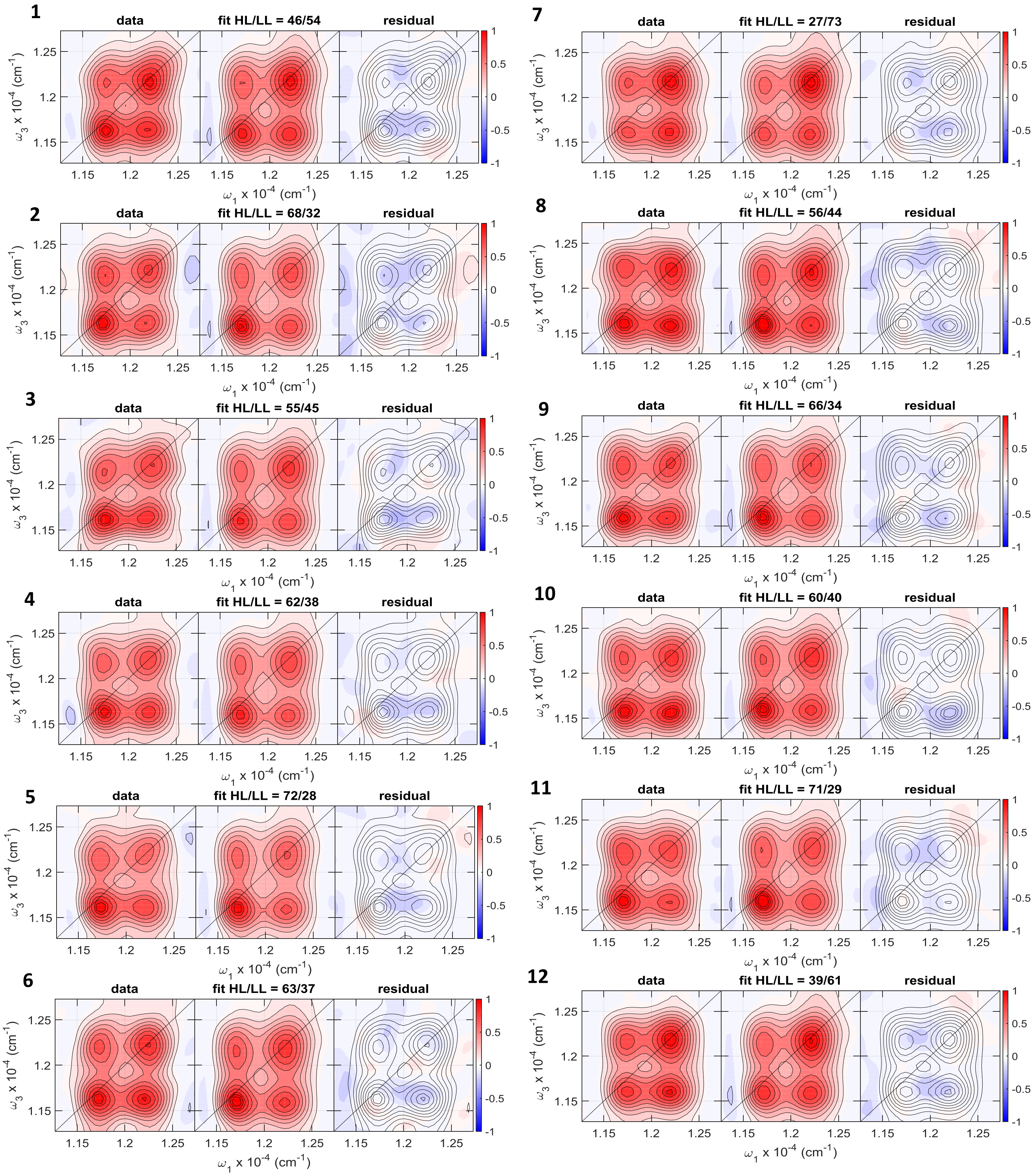

Supplementary Figure 3 (above). SF-2DES spectra corresponding to the lower horizontal array of positions marked by red squares in Fig. 3a and numbered here from 1 (left) to 12 (right).

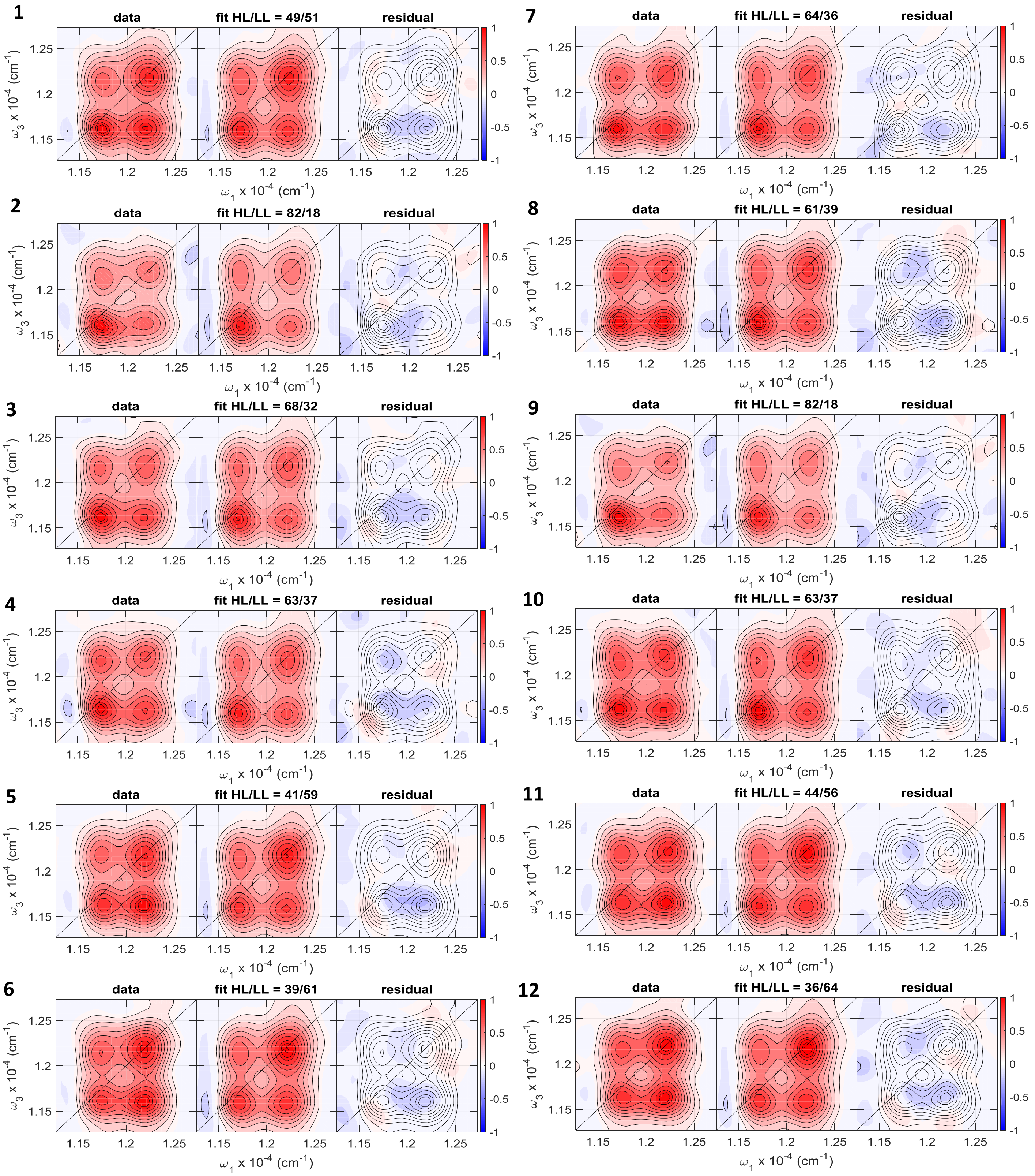

Supplementary Figure 4 (above). SF-2DES spectra corresponding to the middle horizontal array of positions marked by red squares in Fig. 3a and numbered here from 1 (*right*) to 12 (*left*).

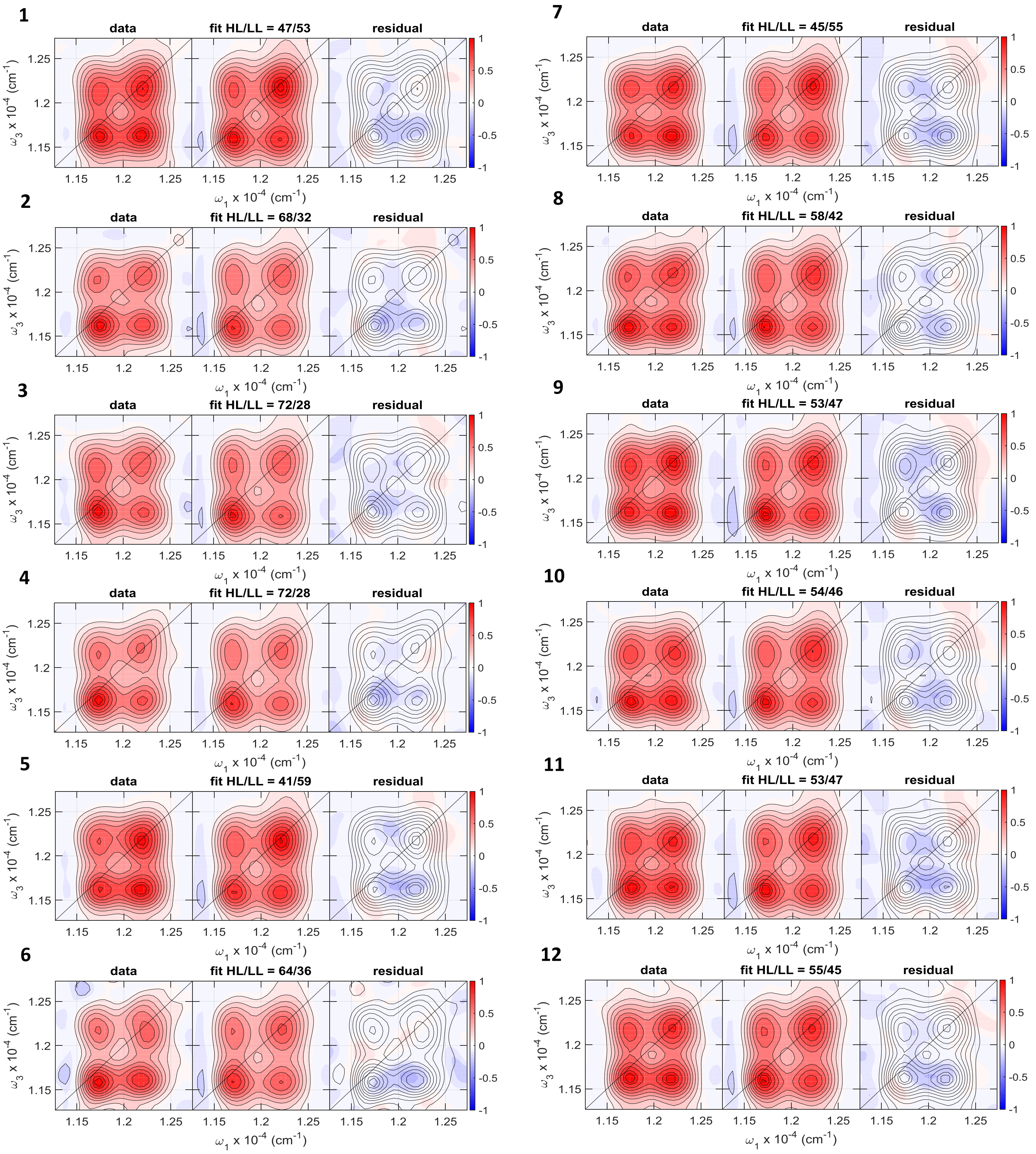

Supplementary Figure 5 (above). SF-2DES spectra corresponding to the top horizontal array of positions marked by red squares in Fig. 3a and numbered here from 1 (left) to 12 (right).

Supplementary Note 2 - Faster Data Acquisition Using Physical Undersampling in SF-2DES

The number of data points required to obtain the full information content of a two-dimensional interferogram is significantly reduced in SF-2DES[1]. The monochromator settings determine the reduced frequencies at which the signals (rephasing and nonrephasing) oscillate. For the data reported here, the monochromator output light was centered at 826 nm (ω_{ref} ~ 2.28 rad/fs). The characteristic excitons in photosynthetic bacteria at 800 nm (ω_{800} ~ 2.35 rad/fs) and 850 nm (ω_{850} ~ 2.22 rad/fs) are downshifted in frequency, $\omega_{800} - \omega_{ref}$ ~ 0.07 rad/fs and $\omega_{850} - \omega_{ref}$ ~ - 0.06 rad/fs, due to physical undersampling[1] in SF-2DES. Taking a step size of 10 fs in t_1 and t_3 corresponds to the Nyquist limit of ~0.31 rad/fs which is ~5x above the Nyquist limit necessary to resolve these excitonic transitions. Therefore, the physical undersampling inherent in SF-2DES allows shorter experimental time and laser exposure in samples susceptible to rapid photodegradation. Supplementary Figure 6 shows that if the data shown in the left panel was collected in 10 fs steps (instead of 5 fs), the data acquisition time will go down proportionally to the number of time points collected, to ~12 seconds, without affecting the locations or relative amplitudes of the 2D peaks. However, "discretization noise" introduced in the data, which is proportional to the $1/N^{1/2}$, where N is the number of sampled data points[2], will also affect the noise floor of the data after a certain step size. Hence, faster acquisition times while having an optimum number of sampled data points is key, the choice of which becomes sample-dependent.

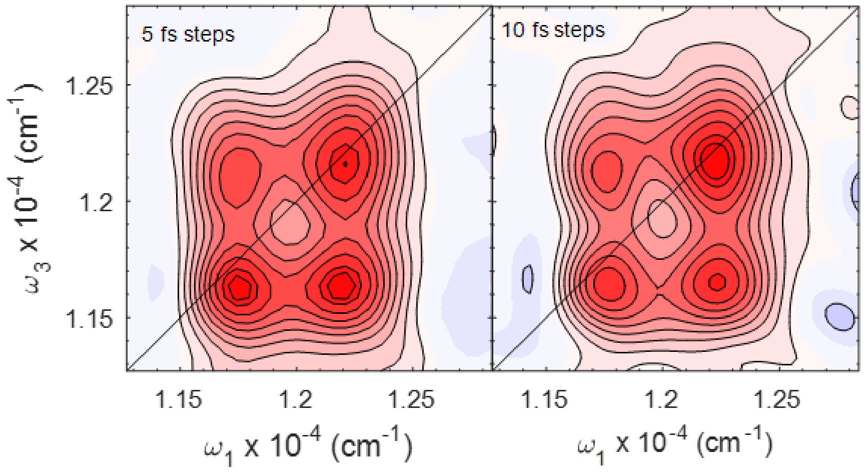

Supplementary Figure 6. Normalized t_2 = 0 fs absorptive 2D spectra collected at the green solid dot location shown on the fluorescence image in Fig 3a. Data points for the 2D spectrum on the left were collected every 5 fs from 0 to 90 fs in t_1 and t_3. The spectrum on the right was calculated using the data set on the left, but ignoring every other data point, such that the effective step size

in t_1 and t_3 is 10 fs. The spectral features, their relative amplitudes and locations are not affected across 5 fs or 10 fs step data sets. The data collection time with 5 fs steps is ~45 secs compared to ~12 seconds which would be needed if the data were collected in 10 fs steps. Contours are drawn at 10-90 % in steps of 10%, with additional contours at 95% and 100% to highlight small differences in maxima. Each spectrum is normalized according to its individual maximum.

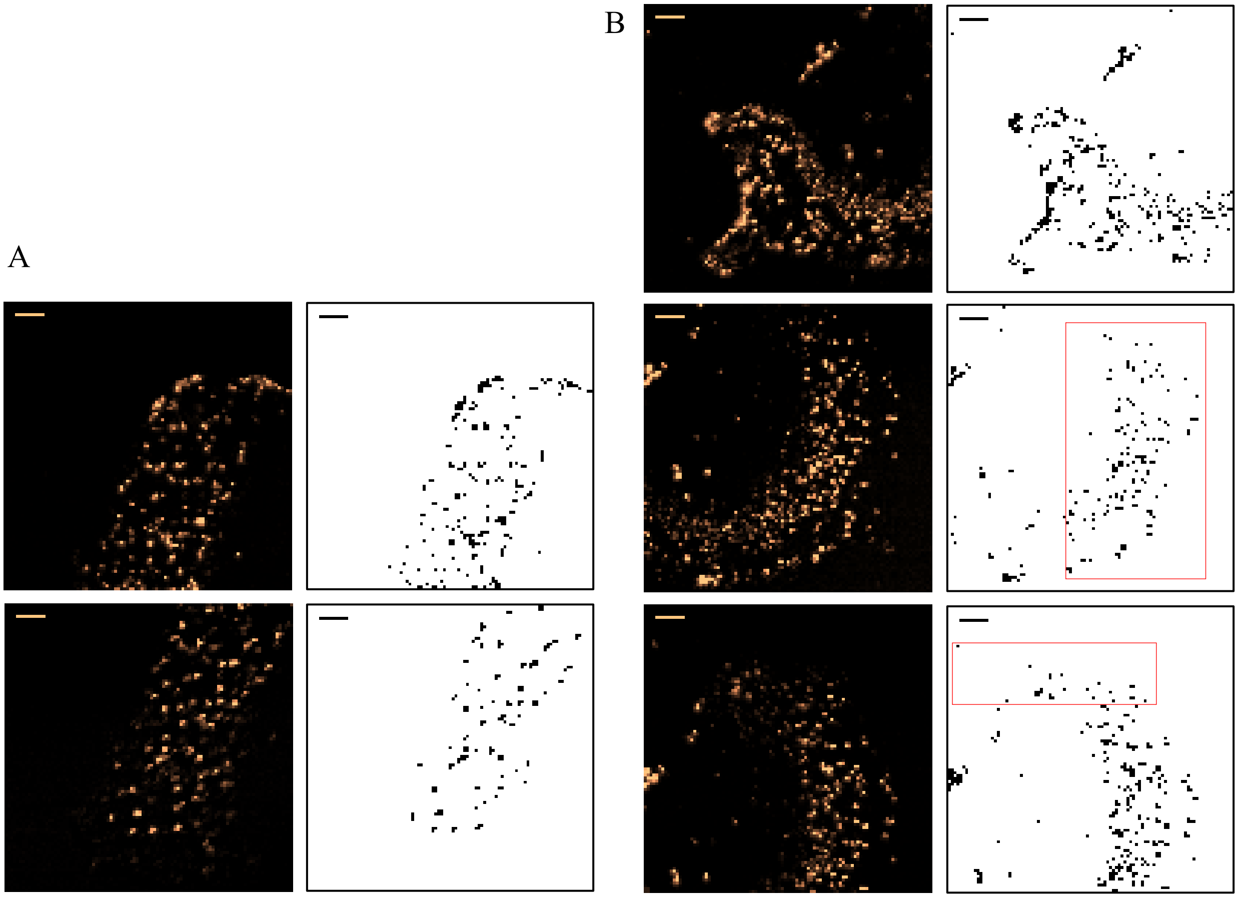

Supplementary Figure 7. Images depicting sections of two dried drops of a diluted solution. An outline mask of each image after thresholding using Otsu's algorithm in ImageJ. **a.** Two sections of the dried layer from a given drop. **b.** Three sections of the dried layer from another drop. The red rectangles correspond to the manually selected region of interest which minimally overlaps with the previous section(s).

Supplementary Note 3 – Estimation of number of cells contributing to the signal

In order to estimate the number of bacteria contributing to the signal we first performed a three steps serial dilution of the original solution amounting to a factor of 10^7. This is a common first step in numerous cell counting protocols looking to obtain a well separated distribution of cells on a surface. Then, small drops of the diluted solution were deposited on the hydrophobic surface of a cover glass using a syringe and left to dry under a Nitrogen atmosphere. The drop volume was estimated to be about 0.08 μL using gravimetric analysis. Drying of a droplet on a hydrophobic surface helps achieve a uniform particle deposition and suppresses ring-like

deposits[3]. Subsequently, the cover glass was sealed using double sided tape and mounted on a piezo positioning stage for confocal imaging. The stage can scan an area of 100 μm x 100 μm. In most cases the layer left by each drop of sample exceeded this area, thus requiring imaging of multiple sections.

In order to count the number of bacteria left by each drop we used a technique similar to that proposed by Zhongli Cai *et al.*[4]which employed freely available ImageJ software for digital cell counting. Each image is initially stored in an 8-bit format with every pixel having 256 values (ranging from 0 to 255) available to display intensity. The first step was to convert the 8-bit format to a binary format based on threshold. This was done using Otsu's automatic threshold algorithm, which efficiently differentiated between the bright features observed in the raw images and the background pixels. The average upper threshold value was about 50 counts, meaning that every pixel ranging from 0 to ~50 was set to zero intensity level (background) during thresholding. In other words, pixels with brightness up to ~20% of the maximum brightness in the image (from ~50 to 255) were considered for counting. In an actual experiment, the brightest 80% fraction is expected to contribute the majority of the FWM fluorescence signal. Subsequently, particles (bacteria) were counted using the "Analyze Particles" functionality, setting the minimum particle size to 1 pixel and the circularity values ranging from 0 to 1. In cases where images overlapped, the appropriate region of interest was manually selected to avoid over counting (Supplementary Figure 3b). In cases were pixels are closely packed together the algorithm counted them as one particle, therefore this method provided an lower bound approximation for the number of bacteria of 138 on average. The upper bound was estimated by counting all pixels above 50 counts on each raw image or those with a brightness up to 19.5 % of the brightest pixel in the image. This way we obtained an approximation of 218 bacteria on average. We therefore estimate that the number of bacteria deposited by each drop of the diluted solution lies on average between 138 and 218. This estimation of the number of cells can be scaled back to the number of cells in a 0.08 μL drop of the original solution using the dilution factor. Assuming ~200 cells per 0.08 μL drop,

Number of cells in 0.08 μL of original cell solution = ~2 x 10^9 cells.

Approximate diameter of the dried drop = ~1 mm.

In order to estimate an upper bound on the number of cells contributing to the signal, we can start by assuming a monolayer of cells making a 1 mm diameter drop. Under such an assumption,

Areal density of cells (in the dried drop) = 2 x 10^9 cells/ (*Area of the dried drop*) = *2550 cells/*μm^2

This represents the *maximum* possible areal density of cells for the ~0.08 μL drop from a solution of a given OD.

Based on Fig. 4b, the maximum lateral area projected by the 10% contour of the non-ideal FWM PSF is ~0.43 μm^2. As assumed above, if the cells form a monolayer, the maximum estimated number of cells will be -

Maximum possible areal density x *Maximum lateral area projected by the 10% contour of the FWM PSF* = 2550 cells/μm^2 x 0.43 μm^2 ~ 1 x 10^3 cells.

Note that even if the cells did not make a monolayer, as long as the thickness of the layer is within the axial resolution of the FWM PSF (Fig. 4b, main text), the resulting estimate of the number of cells contributing to the signal will be an upper bound. This can be seen as follows:

In the case that the monolayer thickness was **H**, the resulting cell density is –

Cell density for a monolayer of thickness **H** μm

= 2 x 10^9 cells / (*Area of the dried drop* x **H** μm)

= (*Maximum Possible Areal Density*)/**H** cells/μm^3.

As an upper bound, the 10% PSF contour could be well approximated by a cylindrical volume in three dimensions. Thus,

Volume of the FWM PSF that probes the sample thickness **H**

= *Maximum lateral area projected by the 10% contour of the FWM PSF* x **H**.

Note that that above quantity increases with the thickness of the sample as long as **H** < = Axial PSF length = 1.3 μm. If **H** > 1.3 μm, the above volume is constant, and is equal to the volume enclosed by the 10% FWM PSF contour, that is, 0.55 μm^3 from Fig. 4b. Thus, out of total number

of cells in the 0.08 μL drop spread homogeneously across 1 mm diameter, maximum number of cells that could contribute to the FWM signal is for the case when the layer thickness is entirely contained within the volume of the FWM PSF, that is, for $\mathbf{H}_{max}$ = 1.3 μm. For **H** > 1.3 μm, the monolayers beyond the axial length of FWM PSF will not contribute appreciably to the total signal. Thus,

Maximum number of cells contributing to 90% of the signal

= (*Maximum Possible Areal Density*)/**H** x *Volume of the PSF that probes the sample thickness* **H**; $\mathbf{H}_{max}$ = 1.3 μm

= ~1 x 10^3 cells

Thus, even though the assumption that the cells dry as a monolayer is most likely incorrect, the estimate on the number of cells contributing to the signal holds because the above expression for the *upper bound* does not depend on the thickness **H** of the layer.

Supplementary Note 4 – Measurement of experimental confocal image of a 0.5 μm diameter bead

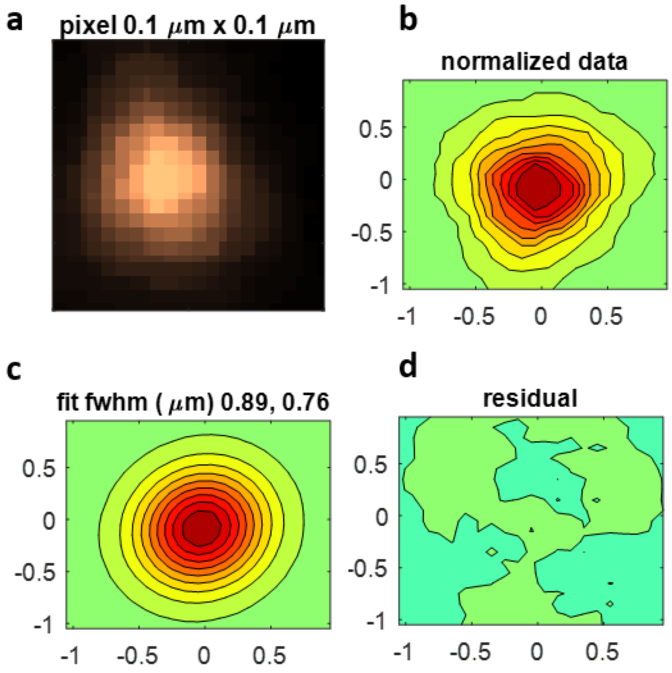

Supplementary Figure 8. **(a)** Experimentally measured confocal fluorescence image from a 0.5 μm diameter fluorescent bead with peak absorption and emission at 763 nm and 820 nm, respectively. In order to collect the fluorescence at 820 nm in a confocal geometry, an OD4 800 SP excitation filter (Chroma) is used along with a 785 nm dichroic mirror (Semrock) and an OD4 815 LP detection filter (Chroma). The peak laser wavelength due to the 800SP filtered excitation is 780 nm. A water objective (60X, NA1.2) was used for imaging. **(b)** Fluorescence image in panel (a), represented as a contour map. **(c)** A rotated elliptical Gaussian fit of the data shown in panel (b). The full width half maxima (FWHM) obtained from the fit are - 0.89 μm and 0.76 μm. **(d)** The residual resulting from the rotated elliptical Gaussian fit of the data. The residual contours lie between 5-10 %, with a residual sum of squares of 0.43. Contours are drawn at 10-100 % in steps of 10 %. Plots in panels (b) – (d) are normalized with respect to panel (b). The average diameter of the bead measured by the lateral confocal PSF in panel (a), and obtained by a rotated elliptical Gaussian fit in panel (b), is 0.83 μm.

The confocal fluorescence image in Supplementary Figure 8 of the text was fitted to a rotated elliptical Gaussian with the functional form –

$$z(x,y) = z_0 + A\exp\left[-0.5\left(\frac{(x - xc)\cos\theta + (y - yc)\sin\theta}{w_1}\right)^2 - 0.5\left(\frac{-(x - xc)\sin\theta + (y - yc)\cos\theta}{w_2}\right)^2\right]$$

In the above equation, the fit parameters are defined as - z_0 is a constant offset, *xc* and *yc* define the center of the Gaussian, θ defines the rotation angle and w_1 and w_2 define the widths of the major and minor axis of the ellipse. The non-linear least squares fitting was performed in MATLAB by minimizing the residual sum of squares using the Levenberg-Marquardt algorithm.

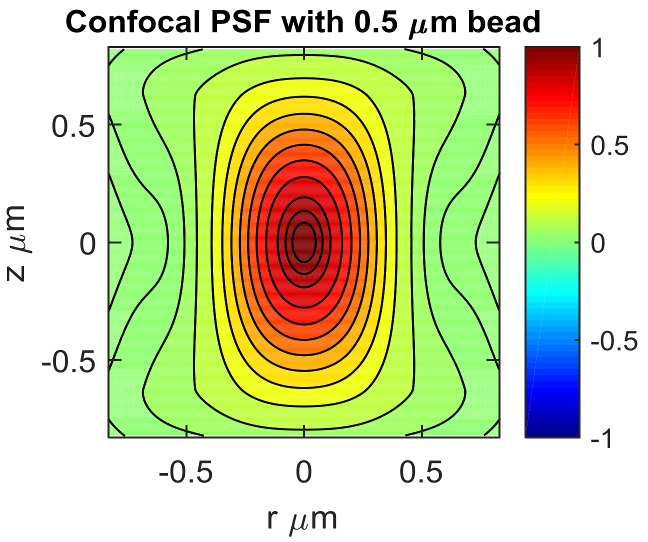

Supplementary Figure 9. Theoretically expected diameter from a confocal image of a 0.5 μm diameter bead. Calculated diffraction-limited confocal point spread function obtained by convoluting the diffraction-limited confocal point spread function of a point source with a spherical bead of 0.5 μm diameter. The peak laser excitation wavelength is 780 nm, and a water objective (60x,NA 1.2) is assumed. The calculation also assumes a large detection pinhole such that H_{conf} (r,z) ~ H_{conv} (r,z) (see main text). The lateral and axial FWHM from the above confocal PSF are ~0.52 μm and ~0.95 μm, respectively. Contours are drawn at 0.1%, 1%, 2%, 5%, 10-90 % in steps of 10%, 95% and 98%. The calculation is based on the equations given in ref.[5] . Comparing the lateral confocal widths with the experimental measurement in Supplementary Figure 8 shows that the deviation between the ideal and measured lateral width is 0.83/0.52 ~ 1.6x.

Supplementary Note 5 – Theoretical point source PSF, and modeling of 1.6x experimentally measured broadening by convolution of point source PSF with a 0.54 μm bead

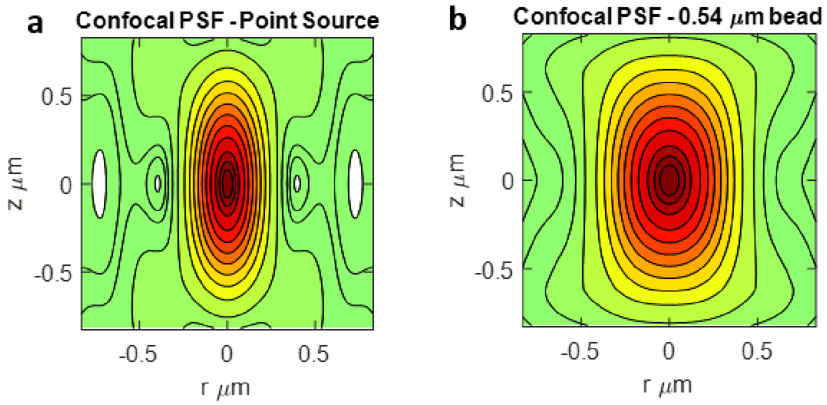

Supplementary Figure 10. (a) Calculated diffraction-limited confocal PSF of a point source. (b) Calculated diffraction-limited confocal PSF obtained by convoluting the diffraction-limited confocal PSF of a point source (panel (a)) with a spherical bead of 0.54 μm diameter. The peak laser excitation wavelength is 780 nm, and a water objective (60x, NA 1.2) is assumed. The calculation also assumes a large detection pinhole such that $H_{conf}(r,z) \sim H_{conv}(r,z)$ (see main text). In panel (a), the lateral and axial FWHM from the calculated confocal PSF are ~0.34 μm and ~0.91 μm, respectively. In panel (b), the lateral and axial FWHM from the calculated confocal PSF are ~0.57 μm and ~0.97 μm, respectively. Contours are drawn at 0.1%, 1%, 2%, 5%, 10-90 % in steps of 10%, 95% and 98%. The calculation is based on the equations given in ref.[5]. Convolution of the ideal confocal PSF with a bead of 0.54 μm approximates the measured (~1.6x) increase in the lateral width of the PSF shown in Supplementary Figures 8-9.

Note that Supplementary Figure 8 and Supplementary Figure 9 compare the measured and expected lateral width from a confocal image of a 0.5 μm bead (0.83 μm measured versus 0.52 μm expected, that is, ~1.6x broader than expected).

Supplementary Figure 10a calculates a point source PSF, and broadens it (Supplementary Figure 10b) by the experimentally measured amount (0.34 μm from point source broadened to 0.57 μm after convoluting with a bead of 0.54 μm). This is achieved by assuming that the experimental broadening can be modeled as a convolution of point source PSF with a 0.54 μm bead.

Supplementary Note 6 – Comparison of Raw versus Zero-padded 2D spectra

Here we compare raw and processed data and show that the data processing steps only account for symmetric changes in 2D spectrum of 10-15% or less. Supplementary Figure 11 (left panel) shows the raw real absorptive 2D spectrum, and the right panel shows the effect of zero-padding the raw data on the distinct 2D cross-peaks. If N points are collected in the experiment along each time axis (19 points from 0 to 90 fs in 5 fs steps in this case), then due to enforced causality in the Hilbert transform (resulting in Kramer-Kronig relation between the absorptive and dispersive parts of the signal), N more data points have to be added to the collected time series[6]. Padding zeros such that the total points are *more* than $2N$ points results in mere interpolation without any increase in resolution as pointed out by Bartholdi and Ernst[6]. The spectrum in the left panel is with $2N$ points, and the right panels is with 128 points.

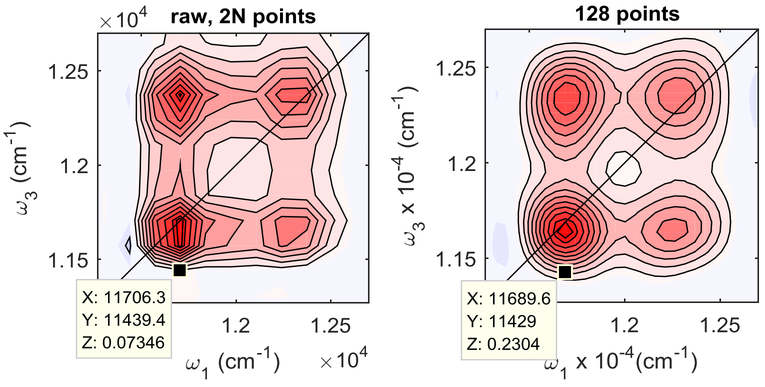

Supplementary Figure 11. Real absorptive $t_2 = 0$ 2D spectra obtained a circulating sample of HL cells. (Left) Without any additional zero padding beyond $2N$ points, where N is the number experimentally collected time points (19 points). As pointed out by Bartholdi and Ernst[6], padding additional N points (total $2N$ points) is a result of Kramer-Kronig relations and *can* lead to an increase in resolution, whereas padding zeros beyond $2N$ results in mere interpolation of the raw data with no increase in spectral resolution along $\omega_{1,3}$. (Right) Zero padding with total 128 points. All figures show a $(X,Y)\equiv(\omega_1,\omega_3)$ data point at ~875 nm along the ω_3 axis, where the laser spectrum is approximately zero. The ‘Z’ value shows the 2D signal level at that location. Comparison of left and right panels shows that due to zero-padding after 90 fs, the 2D amplitude at ~875 nm increases by ~15%.

Supplementary Figure 11 shows that data processing does not change the resolution of 2D peak shapes, but merely interpolates such that in zero padded data (128 points) the contour levels look continuous. The 2D peak shapes slightly broaden but the overall effect of data processing appears

symmetric along both frequency axes. Zero-padding causes the signal level at positions with almost no laser spectrum to be on the order of ~10-15%. Below, we discuss that photo-bleaching related distortions in the 2D spectrum (also shown in Supplementary Figure 1) are the major contributor towards asymmetric 2D peak distortions along ω_3 axis beyond 2D locations inaccessible the laser spectrum.

Figs.2b and 3b in the manuscript show ~20-30% 2D signal at ω_3 locations beyond 875 nm where the laser spectrum is zero. In general, the non-linear fluorescence signal in the fluorescence-detected 2D experiment results from the convolution of the non-linear response function with the 4 electric fields. It follows that, in the frequency domain, the effect of the laser spectrum can be simply understood as creating a frequency filter on the non-linear susceptibility[7]. Supplementary Figure 12 (and accompanying caption) shows that *asymmetric* distortions and associated signal increase beyond 875 nm are accounted for by photo-bleaching (as also shown in Supplementary Figure 1 in the SI).

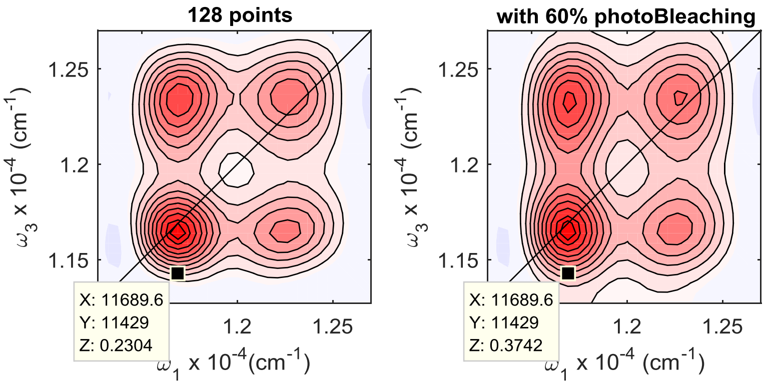

Supplementary Figure 12. (left) Same 2D spectrum as shown in Supplementary Figure 11 right panel. The sample is circulated for obtaining this spectrum such that any photo-bleaching is negligible. (right) 2D spectrum on the right but with a simulated 60% signal photo-bleaching rate (see description of Supplementary Figure 1 in the SI). The marked location corresponds to ~875 nm on ω_3 axis where there is no laser spectrum, but the 2D signal shows vertical distortions leading to ~15% signal increase at 875 nm, compared to the spectrum on the left. As described in Supplementary Figure 1, asymmetric vertical distortions occur because the scanning scheme used in the experiment scans t_1 delays first and t_3 delays last.

Supplementary Note 7 – Estimation of fluorescence-detection sensitivity, and low-repetition rate experiments

Mie scattering background from the cells makes *in vivo* conventional 2DES studies very challenging and susceptible to ghost peaks[8]. In contrast, fluorescence 2D provides facile color filtering of the signal, and therefore an attractive route to *in vivo* studies. Below we compare our approach to the competing approaches in conventional 2DES which have demonstrated *in vivo* studies[9-11]. Given the differences in focusing, repetition rate, as well as the different detectors and the need for scatter subtraction in conventional 2DES it is very difficult to isolate the effect of fluorescence detection alone. Studying a variety of scattering samples with both methods, keeping as many other factors as equivalent as possible, can be done to address this subject in greater detail. We performed experiments at lower repetition rates (data shown in Supplementary Figure 13) to rule out artifacts in our spectra that could be caused by the use of high repetition rates. This data, which was not spatially resolved, was acquired with focusing conditions and optical densities closer to those used in a conventional 2DES measurements and enables a rough estimate of the relative sensitivity of fluorescence vs conventional detection. Below we compare some of the relevant experimental parameters of our work and other *in vivo* 2DES studies[9-11].

Supplementary Table 1 compares the experimental parameters used in our *in vivo* 2D studies, both the SF-2DES measurements reported in the main paper text (column 2) and lower repetition rate non-spatially-resolved measurements (F-2DES) shown in Supplementary Figure 13 (column 3). Note that the study by Zigmantas and coworkers in the last column is on green sulfur bacteria, and is performed at 77 K, whereas the other studies have been performed at 300 K on circulating purple bacterial samples. Room temperature studies on circulating samples suffer from significantly higher scattering background and lower signals at 300 K. Therefore, directly comparing the SNR from ref.[9] to SF-2DES or the studies by Engel and co-workers[10,11] will not be a fair comparison. Note that for the approach of Engel and coworkers[10,11], a given point on the 2D spectra during the scan will be $(t_{1,}\lambda_3)$, whereas for SF-2DES it will be (t_1, t_3). For both such points, the total number of laser shots required to generate a final absorptive 2D spectrum have been considered. Even though the approach of refs.[10,11] can in principal generate a 2D spectrum per laser shot, the scatter removal process significantly slows down the acquisition time.

	Ref. [10,11] (Engel and co-workers)	SF-2DES (Figure 3c of main paper)	F-2DES (Supplementary Figure 13 of SI)	Ref. [8,9] (Zigmantas and co-workers)
Repetition Rate	5 kHz	83 MHz	500 kHz	20 kHz
per pulse energy (nJ)	1.4	9E-5	0.003	1
Fluence (μJ/cm^2/pulse)	17.6	12.4	0.75	12.7
Sample OD (200 μm)	0.3	0.06	0.3	0.2
Scatter Subtraction	YES	NO	NO	YES
Total Laser Shots per point on the final 2D Spectrum	*100 laser shots* per point (25 Hz) x average of *500 points around a given T* x *3 trials* ~ **1E5 shots**	*20 ms lock-in acquisition time per t1,t3 step* x *83MHz* ~ **1E6 shots**	*580 ms lock-in acquisition time per t1,t3 step* x 500 kHz ~ **2.9E5 shots**	NA
Total Acquisition Time per Absorptive 2D	20 seconds	12 seconds with 10 fs t1,t3 steps	70 seconds with 10 fs t1,t3 steps	NA
Lock-in Detection	NO	YES	YES	YES
Temperature	300 K	300 K	300 K	77 K
Pump-probe Focal Volume (μm^3)	~10^6	~1	~10^6	~10^6

Supplementary Table 1 – Comparison of SF-2DES approach with the *in vivo* 2D studies on purple bacterial cells from Engel[10,11] and co-workers at 300 K and *in vivo* 2D studies from Zigmantas[9] and co-workers at 77 K. We also include fluorescence-detected 2DES (F-2DES) at lower repetition (500kHz) and focusing conditions closer to those used in conventional 2DES measurements. Refs. [10,11] collect a 2D spectrum per laser shot (without any mechanical scanning), but average finely around each T which is required in order to remove the scatter background. Note that the details in refs.[8,9] were insufficient to confidently estimate the total data acquisition times. Refs.[8,9] use mechanical wedge pairs in order to scan the t1 and t3 time delays. However, due to amplitude modulation and lock-in detection, fine T scanning is not required to remove the scatter. Hence, the total data collection times required to generate a processed 2D spectrum should be of

the order of a few seconds and similar to refs.[10,11]. Experimental parameters which are the most different between the approaches are highlighted in green.

Comparing Supplementary Figure 13 with Fig. 1 of ref. [11], we assert that the SNR in Supplementary Figure 13 is comparable if not better than ref. [11]. Based on a comparison of columns 1 and 3 in Supplementary Table 1, since the four-wave mixing signal is proportional to pump and probe fluences, and square root of total number of laser shots, the SNR reduction in F-2DES compared to conventional 2DES should have been $SNR \approx \sqrt{2.9/1.5}(0.75/17.6)(0.75/17.6) \approx 0.0025$. Based on the above assertion, we estimate that the sensitivity of fluorescence detection is at least 1/0.0025 ~ 400x better in the F-2DES case. Note that the above factor does not decouple the effect of scatter from the sensitivity of fluorescence detection and we expect that the relative sensitivity of F-2DES and conventional 2DES will depend strongly on differences in sample scattering as well as fluorescence quantum yield. We note that further improvement could be made for the fluorescence-detection approach here by more efficiently-collecting the signal. For the measurements in Supplementary Figure 13, a numerical aperture of ~0.4 was used for the signal collection. Thus an additional ~3^2x improvement[12] could be obtained with the use of a 1.2 numerical aperture objective, making the expected improvement ~3600x.

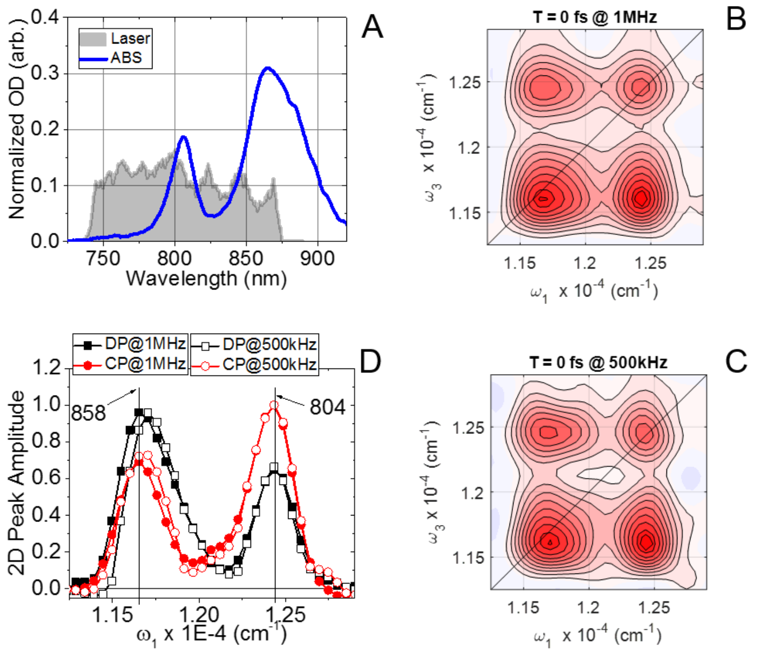

Supplementary Figure 13. (A) 1MHz/500kHz laser spectrum overlaid with the background subtracted HL cells linear absorption spectrum. (B, C) Absorptive t_2 = 0 fs F-2DES spectra for

1MHz (B) and 500 kHz (C) laser repetition rates. (D) Diagonal peak (DP) and cross-peak (CP) slices corresponding to 1 MHz (solid) and 500 kHz (hollow). The DP and CP peak positions in nanometers are marked in the figure. Supplementary Figure 13B,C show 2D spectra from circulating HL cells with OD ~0.3, and a ~16 μm focal spot size obtained from a 5 cm lens (instead of ~1 μm from the microscope objective). The laser fluence incident at the sample is 1.5 μJ/cm^2/pulse for 1 MHz and 0.75 μJ/cm^2/pulse for 500 kHz repetition rate. *Thus, compared to the 2D spectra reported in the manuscript, the measurements shown in Supplementary Figure 13 correspond to a combination of 100x lower repetition rates, 16x bigger focal spot size, and 16.5x lower pump and probe fluences*. The spectra represent the lowest fluence for which a respectable signal-to-noise ratio (SNR) was attainable without substantially longer acquisition times compared to before. The distinct cross-peaks at t_2 = 0 fs persist even under contrastingly different fluence, repetition rate and focusing conditions. For these repetition rates and fluences, Kohler and co-workers estimate[13] <3% steady state carotenoid triplet population in detergent-isolated LH2 complexes. In the presence of neighboring LH2s and LH1-RC 'sink', the carotenoid triplet fraction is expected[13-15] to be even lower in LH2s, and exist predominantly on LH1-RC complexes (see section "Modeling of Repetition Rate Dependent Populations" in the SI). Supplementary Figure 13D shows that the cross-peak (CP) and diagonal peak (DP) slices through the peak maxima for the two repetition rates are in decent agreement. Notice that due the flatter laser spectrum compared to the 83 MHz source used in the main paper, the position of the B800 band is closer to the expected position of 805 nm, in agreement with the linear absorption spectrum. As discussed in response to comment 6, the relative DP and CP peak amplitudes are sensitively dependent on the pump and probe laser intensity at the B800 and B850 bands, and the amount of GSB, ESE and ESA contributions at each peak at t_2 = 0 fs.

<u>Experimental Details for Supplementary Figure 13</u>

A white light continuum is generated by focusing the 1 MHz (or 500 kHz) 1040 nm output of a tunable repetition rate laser amplifier (Spectra Physics, Spirit 1040-16) into a 4 mm YAG crystal, followed by removing the fundamental with two OD6 630 nm long pass optical filters (Chroma, E630LP). The resulting beam is collimated and routed into the experimental setup reported in the manuscript. The spectrum is further cleaned through OD6 740 long pass (Chroma) and OD4 875 short pass (Edmund) optical filters. Note that the output of the SF-2DES spectrometer is not routed into the microscope objective. Instead, a 5 cm lens focuses the pulses (16 μm FWHM spot size, 13 fs FWHM pulse duration) into a 1 mm pathlength flow cell (Starna 584.4-Q-1), through which the sample is recirculated at ~260 ml/min using a peristaltic pump. The fluorescence is collected at 90 degrees with optics possessing a numerical aperture of ~0.4, and isolated using two OD6 887 long pass filters (Semrock).

Supplementary Note 8 – Comparison of SF-2DES with other spatially-resolved 2DES approaches for imaging the entire sample

The fluorescence images shown in the paper are collected with a 5 ms binning time for each X,Y location of the piezo stage. For a ~2 μm step size which is comparable to the current resolution of SF-2DES spectrometer (~1μm FWHM), the total time for collecting a 100 μm x 100 μm fluorescence image is ~0.005x2500 ~ 13 seconds.

The 2D spectra reported in the manuscript took ~45 seconds. However, Supplementary Figure 6 shows that 2D spectra with similar signal to noise and similar 2D peak shape resolution can be collected with 10 fs $t_{1,3}$ steps in ~ 12 seconds. Thus, for collecting 2500 successive 2D spectra, the total imaging time required will be ~2500x12secs = 8.3 hours. Previously, we have utilized the SF-2DES spectrometer for collecting 2D on circulating samples for successive waiting times such that the setup exhibits good phase stability over an experimental duration of 4 hours. Hence, collecting 2D spectra over a period of 8 hours is not unfeasible.

In comparison with spatially-resolved 2DIR approaches, the data acquisition time per 2D spectrum in case of the SF-2DES spectrometer are faster than those reported by Zanni et al. (55 secs per 2D versus 12 secs) and Tokmakoff et al. (100 secs per 2D versus 12 secs). However, unlike the parallel wide-field imaging approach adopted by Zanni et al. (ref. 40 in the manuscript), our sequential approach is similar to the one implemented by Tokmakoff et al. (ref. 41 in the manuscript). In case of Zanni et al. due to parallel image collection from multiple locations on the sample, imaging the entire sample is consequently much faster. However, we argue that in the presence of fast photo-bleaching rate in *electronic* spectroscopy, faster data collection at any given point in the sample assumes higher priority than imaging the entire sample in parallel, but with slower data collection times per 2D spectrum. Note that the fluorescence-detected 2D approach by Brixner et al. (ref. 42 in the manuscript) requires a 27 step SSLM based phase cycling with ~ 1.2 seconds for each phase cycle per time step. Hence the 2D spectra collection times in that approach will be considerably slower (~18 mins per 2D spectrum) than the approaches compared here.

We expect that practical applications of our method will likely involve the rapid collection of a fluorescence image for sample evaluation, followed by the acquisition of 2D spectra at selected locations.

Supplementary Note 9 – Absolute calibration of SF-2DES signals to determine number of HL to LL cells contributing to 2D signal from a given spatial location

This approach described below could be used in the future experiments to determine the absolute number of cells which contribute to the measured four-wave-mixing signals. Note that this approach is different from the simultaneous determination of concentration and extinction coefficient performed in ref. [16] on a fluorescein molecule, in that only concentration can be determined.

The idea of absolute calibration for all optical concentration determination has been pursued in recent studies such as absolute pump-probe signal size measurement without any *in situ* calibrant by Jonas and co-workers[16,17], as well relative calibration of 2D spectra using an *in situ* calibrant by Zanni and co-workers[18]. The experimental method and theory outlined in ref.[17], requires calibration of measured signal in terms of absolute number of photon number changes in a pump-probe signal. This requirement is complicated in *in vivo* measurements of fluorescence-detected signal on account of varying fluorescence quantum yield between HL and LL cells. Additionally, although the presented framework[17] is more general, in order to express the unknown concentration in terms of the measured non-linear signal[16], simplifying approximations such as a two-level system, Condon approximation, vibrationally relaxed non-linear signal are used, which will fail for LH2 complexes. Alternatively, a simplified two-step calibration approach that can work in SF-2DES experiments is as follows.

Firstly, we would need to calibrate the measured fluorescence-detected 2D signal (in Volts), for a given amount of LL and HL cells through separate measurements on samples with varying amount of cells. All the signal processing settings have to be kept constant between the HL and LL samples, and all measurements for each kind of sample. By calibrating the measured signal for a known absolute number of cells, the HL and LL *signal ratios* derived from the experiments could be easily calibrated to HL to LL *cell ratios*. Note that this approach assumes

that there are no differences between the cell sizes, number of LH2 complexes within the cells, and fluorescence quantum yields for a given type of cells (HL or LL). Additionally, in the outlined approach, only concentration determination will be possible, not determination of extinction coefficient as was done in ref. [16]. The cell sample is a thick and sticky aliquot, making gravimetric analysis a more viable approach to determine the absolute weight of cells instead of absolute number of cells contributing to the signal. A known weight of cells can be dissolved to make a solution of a constant volume (and therefore a given density of cells). By varying the weight of cells dissolved, a calibration curve (‘Curve A’ in Supplementary Figure 14) can be obtained. The resulting calibration will be separate graphs of *measured four-wave-mixing signal (V) versus density of cells in the prepared sample (grams/cm^3)* (‘Curve A’) for HL and LL samples. Since the probed focal volume will be constant across experiments, the density of cells in solution can be easily converted to weight of cells contributing to the measured signal. Based on ‘Curve A’, the four-wave-mixing signal measured from a mixed sample can first be isolated into HL and LL signals, and then converted to total weight of HL or LL cells which contributed to the signal.

Secondly, in order to convert the density of cells to absolute number of cells, a cell counting approach similar to the one performed in Supplementary Figure 7 can be used. For each of the HL or LL samples used to obtain ‘Curve A’ (each point on ‘Curve A’), a dilution of a known amount, followed by fluorescence imaging can be used to convert obtain ‘Curve B’ where the Y axis will be the *number density of cells cm^{-3}* and the X axis is *density of cells (g/cm^3)* in the diluted samples. With a combination of calibration curves A and B, the amount of measured four-wave-mixing signal can be converted to the absolute number of cells contributing to the signal.

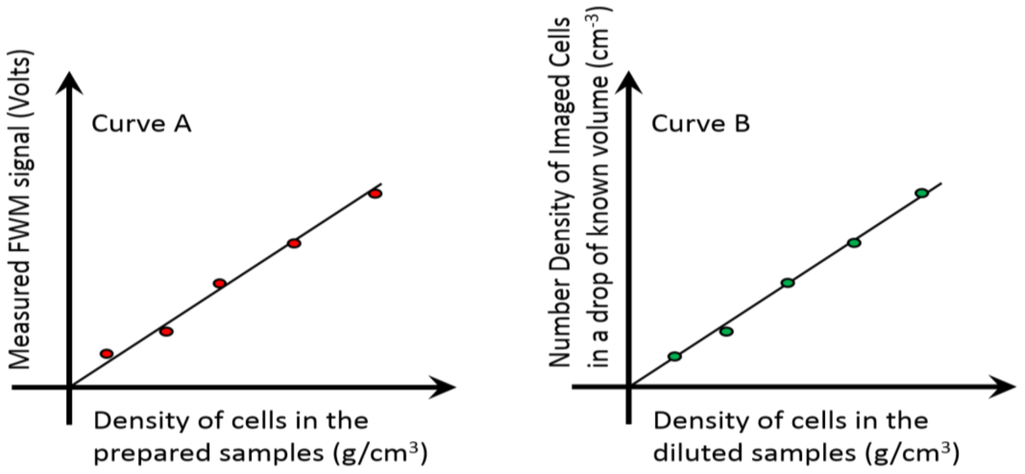

Supplementary Figure 14. Calibration curves A and B which can be used to convert the measured HL/LL FWM signal ratio to HL/LL number of cells ratio. Each point on the calibration curves corresponds to a sample prepared with a known weight of cells (which is varied across different points). For curve B, the samples prepared for curve A are diluted by a known amount which allows easy identification of individual cells in a fluorescence image. For making the imaging samples, a drop of a known volume is dispensed on the coverslip, and the number of cells in that volume are counted through the fluorescence image, resulting in Curve B. A pair of such curves will be required for each kind of sample (HL and LL).

Supplementary Note 10 – Effect of structured laser spectrum on growth condition dependent 2D peak amplitudes and positions

Below we analyze the reported growth dependent differences in the 2D spectra in greater detail and show that –

i) 2D spectra recover growth dependent differences above the error bar of the measurement.

ii) When analyzing the *relative* differences between HL and LL cases, peak positions are strongly affected by the laser spectrum. We argue that with a laser spectrum that covers the B850 band better, the recovered differences will be similar although determined with better accuracy.

With regard to the cross-peak amplitudes, the effect of the laser spectrum is approximately normalized out as long as the pump and probe laser spectra are identical. However, the diagonal peak amplitudes will be strongly affected by the laser spectrum. Additionally, the 2D peak positions will be a weighted product of the laser amplitude with the sample absorption cross-section. We analyze these in detail below.

In *R. palustris*, the B800 band is known to be unaffected by growth conditions, and the most noticeable effect of growth conditions occurs on the B850 rings[19]. There is a weak band at ~810 nm that originates under LL conditions[20], and its intensity is increased under higher 'light stress'. Comparing to ref.[19], the LL cells in the present study correspond to intermediate LL conditions (termed as 'LL1' in ref. [19], and 'lower light' LL in the sample preparation description in the main text). Under intermediate LL conditions, the weak ~810 nm band will be overwhelmed by the B800 band peaking at ~805 nm (for example, see Fig. 1 of ref.[19]). In order to consider the growth dependent differences, and the effect of the laser spectrum on the relative amplitude of

diagonal peaks, we first remove the rising Mie scattering background in order to extract the absorption strengths in the B800 and B850 bands using a single exponential function to model the background as shown in Supplementary Figures 15A,B.

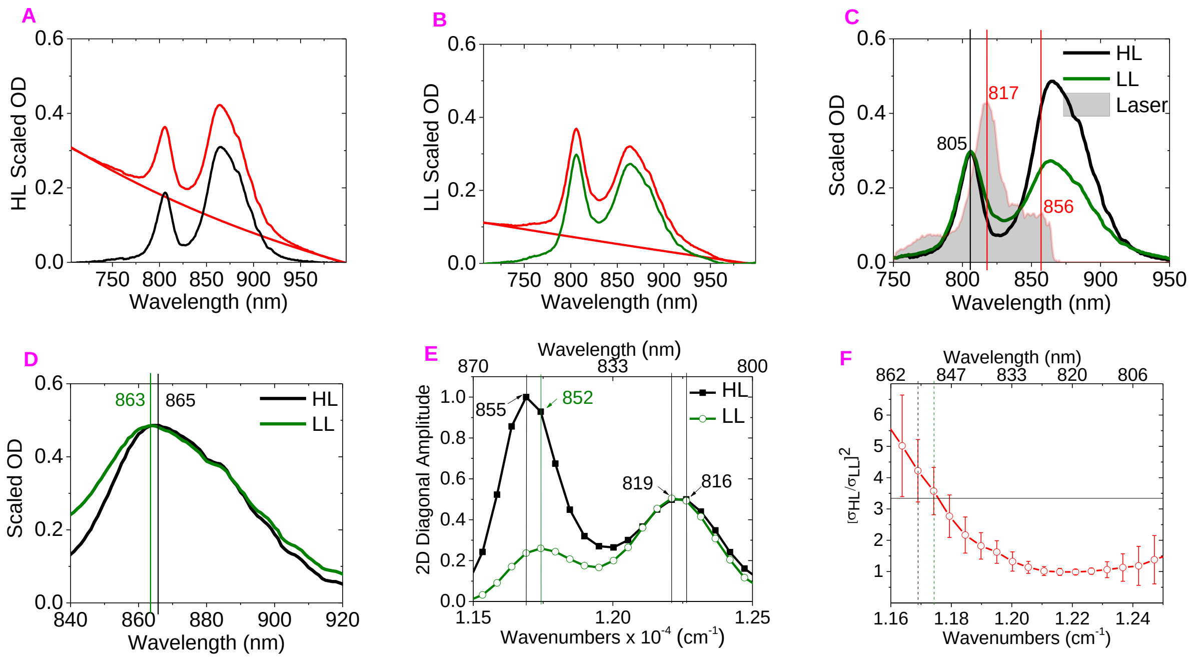

Supplementary Figure 15. Removal of scattering background from the colloidal HL **(A)** and LL **(B)** cell solutions using a single exponential decay as the background function. **(C)** The resulting background free LL and HL cell absorption spectra plotted with the laser spectrum. The absorption spectra are normalized to the B800 band. It can be seen that the B850 absorption strength for the LL case goes down by ~1.8x. Correspondingly, the B800 band appears broader in the LL case due to a weak underlying band at ~810 nm which arises from certain *BChl a* molecules *within individual LH2 rings* getting blue-shifted in site energy under LL conditions[19]. The approximate peak positions of the B800 band, laser spectrum, and the red shoulder of the laser spectrum are marked on the figure. **(D)** Same absorption spectra as panel C, but normalized relative to the B850 band. Upon normalization, it is seen that the B850 band for LL case, appears broader and ~2 nm blue shifted. The shift is of the order of the resolution of the absorption spectrum (1 nm) and should be considered very approximate. **(E)** Diagonal 2D amplitudes for HL and LL cells (same as Fig. 2D in the manuscript but without the error bars). The 2D diagonal peak positions are marked. Comparison with the B800 band position from the absorption spectrum (805 nm from panel C) shows that the 2D diagonal peak position for both HL and LL cells is red-shifted to ~818 nm due to the laser spectrum peaking at ~817 nm. Similarly, the diagonal peak corresponding to the B850 band, which peaks at ~863-865 nm in the linear absorption spectrum (panel D), now peaks at 852 nm for LL and 855 nm for HL cells, close to the peak of the red shoulder of the laser spectrum (panel C). The slight blue shift in the LL case is most likely related to the broadening and ~1-2 nm blue shift seen in the linear absorption spectrum (panel D). **(F)** Ratio of the HL and LL 2D diagonal amplitudes from panel E, along with the propagated error bars. The ratio at ~816 nm is 1 because the bands are normalized with respect to the B800 diagonal peak amplitude. The dashed lines correspond to the peak positions of the HL and LL curves shown in panel E, 855 nm and 852 nm

respectively. The ratio at 855 nm and 852 nm is 4.2(±0.9) and 3.6(±0.7), respectively. The horizontal solid black line corresponds to the theoretical HL/LL ratio of 3.3, of the B850 absorption strength derived from panel C for a vibrationally relaxed 2D spectrum.

Supplementary Figures 15C-E show the effect of the laser spectrum on the peak positions of B800 and B850 bands. In the absorption spectrum (Supplementary Figure 15C), both HL and LL B800 bands peak at 805 nm, with the LL band being slightly broader due to the underlying weak ~810 nm band. In the 2D spectrum (Supplementary Figure 15E), the diagonal peaks for HL and LL, both peak at ~818 nm. The B800 band for the LL case is marginally red-shifted (within the resolution of the measurement), possibly due to the broader B800 band (in Supplementary Figure 15C). In the linear absorption spectrum, the B850 band for the LL case is also slightly broader and blue shifted by 1-2 nm (Supplementary Figure 15D) and peaks at 863-865nm. In comparison, the red-shoulder in the laser spectrum peaks at ~856 nm. Consequently, the B850 2D diagonal peak is located between 855 nm for HL and 852 nm for LL case (Supplementary Figure 15E).

If the laser spectrum is given by $i(\omega)$, then, for a vibrationally relaxed 2D spectrum, the 2D absorption strength along the diagonal will be approximately given by a square of the product of laser spectrum and the absorption cross-section, that is, $A_{diag}^{2D} \approx \left(\sigma(\omega)i(\omega)\right)^2$. Therefore, the ratio of HL and LL diagonal peak amplitudes will be given by $A_{diag}^{2D,HL} \big/ A_{diag}^{2D,LL} \approx \left(\sigma_{HL}(\omega).i(\omega)\right)^2 / \left(\sigma_{LL}(\omega).i(\omega)\right)^2 = \left(\sigma_{HL}(\omega) / \sigma_{LL}(\omega)\right)^2$. Note that this ignores any contributions on the 2D peaks caused by ESE or ESA signals, which can occur on the red-side along the detection axis. Theoretically, this *ratio* of diagonal peak strengths should not be affected by the laser spectrum, as long as the laser spectrum for HL and LL measurements is identical. However, a strong gradient in the absorption and laser spectrum, or a zero intensity laser spectrum will affect the experimentally derived ratio and its accuracy. Based on Supplementary Figure 15C (for normalized B800 peak strengths) and the above expression, a ratio of ~3.3 is expected for the B850 diagonal peak strengths derived from a vibrationally relaxed 2D spectrum. From Supplementary Figure 15F, the experimentally measured ratio is 4.2(±0.9) and 3.6(±0.7) at 855 nm and 852 nm, respectively. Since, error bars are proportional to the gradients of the parameters on which they depend, both, the gradient on the red shoulder of the laser spectrum, as well as the gradient in the absorption spectrum at ~855 nm, contribute to large error bars on the ratio. Although within the large error bars, the experimentally derived ratio overshoots the ratio on the red edge of

the measured B850 peaks. We note that the ratio of ~3.3 is only expected from a vibrationally relaxed 2D spectrum after ignoring any red-shifted ESE or ESA contributions, whereas we compare the expected ratio to a t_2 = 0 fs 2D spectrum. Hence, some deviations are expected.

In the reported measurements, the B850 band which has the most visible growth dependence, but is only partially (<50%) probed by the laser spectrum. The 2D peak amplitudes still recover substantial growth dependent differences with large error bars, although the differences seen between B800 and B850 manifolds (between the HL and LL cases) are still easily above the error bar of the measurement. Based on the above analysis, peak positions are more strongly affected by the laser spectrum than the ratio of 2D peak amplitudes. With a flatter laser spectrum (such as that used in a linear absorption spectrometer), the recovered 2D peak positions are expected to be closer to the positions in the linear absorption spectrum. Additionally, absence of a strong gradient in the laser spectrum with the absorption band of the sample will increase the accuracy of the measured peak amplitude (and the relative ratios).

In Fig. 2b in the manuscript, the HL and LL spectra we normalized differently in order to emphasize the growth-dependent differences. That is, the HL 2D spectrum is normalized relative to the B850 diagonal peak (DP_B850), and the LL 2D spectrum is normalized with respect to the B800 diagonal peak (DP_B800). The different normalization is better seen in Supplementary Figure 16 and Supplementary Table 2 below. Supplementary Figure 16 shows the DP and CP slices from Fig. 2b in the manuscript along with the error bars. Supplementary Table 2 shows the peak amplitudes and error bars seen from Supplementary Figure 16. As seen from Supplementary Table 2, normalizing the peak amplitudes with respect to the *DP_B850 for HL* and *DP_B800 for LL* cases, causes the CPs to exhibit less variation in amplitude between HL and LL cells (CP amplitudes between HL and LL are within ~10-15% of each other). In general, the cross-peak strengths are less perturbed than the diagonal peaks because they depend on the product of B800 and B850 absorption strengths.

If the 2D peaks were normalized to the B800 band for both LL and HL cases, then the CP amplitudes for HL would be ~1.2 compared to 0.74 and 0.54 CP amplitudes for the LL case. Similarly, the DP_B850 amplitude will be ~2 compared to 0.51 for the LL case. As mentioned in the discussion around Supplementary Figure 15, these changes are expected and are qualitatively

consistent with the changes seen in the absorption spectra for LL and HL cells (Supplementary Figure 15C).

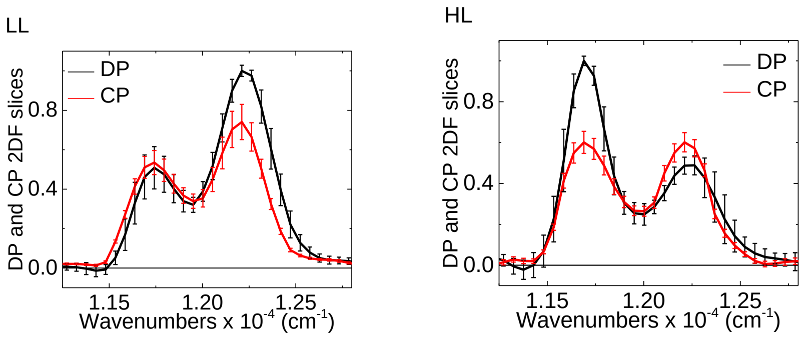

Supplementary Figure 16. 2D DP and CP slices from Fig. 2b in the manuscript with the corresponding error bars. The peak amplitudes are normalized with respect to the *DP_B850 for HL* and *DP_B800 for LL* cases.

NON-IDEAL LASER	Amp HL	Amp LL
DP_B850	1.0	0.51(0.11)
DP_B800	0.49(0.09)	1.0
CP12	0.60(0.05)	0.74(0.09)
CP21	0.60(0.05)	0.54(0.06)

Supplementary Table 2. 2D Peak Amplitudes from Supplementary Figure 16. The corresponding error bars are shown in parenthesis.

In the above discussion, we considered the effect of the laser spectrum on cross-comparisons between the HL and LL 2D spectra. As noted above, the peaks in the 2D spectrum will be proportional to a product of the absorption cross-section weighted by the laser spectrum. For example, for population contribution to 2D peaks are roughly given by -

$$DP\_B800 \approx \sigma_{B800}(\omega) i_{B800}^{Pump}(\omega) . \sigma_{B800}(\omega) i_{B800}^{Probe}(\omega)$$
$$DP\_B850 \approx \sigma_{B850}(\omega) i_{B850}^{Pump}(\omega) . \sigma_{B850}(\omega) i_{B850}^{Probe}(\omega)$$
$$CP12 \approx \sigma_{B800}(\omega) i_{B800}^{Pump}(\omega) . \sigma_{B850}(\omega) i_{B850}^{Probe}(\omega)$$
$$CP21 \approx \sigma_{B850}(\omega) i_{B850}^{Pump}(\omega) . \sigma_{B800}(\omega) i_{B800}^{Probe}(\omega)$$

CP12 is the lower and CP21 is the upper cross-peak. $i_{B800}^{pump}(\omega)$ is the pump laser intensity at the location of B800 band. The above equations suggest that, in the absence of other contributions, such as the ~810 nm band for LL cells, or imbalanced GSB, ESE and ESA contributions between

the two cross-peaks, the cross-peaks will be balanced for identical pump and probe spectrum. However, possible imbalances in the pump and probe intensities at the B800 and B850 locations due to change in experimental conditions can result in relative ratios of CPs as well as DPs to vary accordingly. Thus, cross-comparisons between HL and LL 2D peak amplitudes depend critically on the laser spectrum, and are only valid under identical experimental conditions. For this reason, all 2D spectra reported on immobilized samples (Fig. 2b and Fig. 3b in the manuscript) were collected on the same day with only the sample being switched between HL, LL or mixed. Thus, cross-peak imbalances shown in Supplementary Table 2 arise due to features intrinsic to the LL sample compared to the HL sample. Possibly the ~B810 contributes to this imbalance on the LL sample. Higher ESE and ESA contributions on the lower cross-peak, and vibrations which are strongly coupled to electronic states, through Huang-Rhys factors or non-adiabatic vibrational-electronic mixing[21], can also cause imbalance between the CP amplitudes. Vertical distortions in the 2D spectrum caused by photo-bleaching, as seen in Supplementary Figure 1 in the SI, account for ~5% or less of the imbalance between relative CP peak amplitudes.

The 2D measurements on the circulating samples (Fig. 3c in the manuscript) were collected on a different day than the ones on immobilized samples. Consequently, we do not use Fig. 3c for any analysis between HL and LL samples, and only show it for the purpose of contrasting the noticeable vertical distortions seen in the immobilized samples. Both imbalance between pump and probe spectrum, and photo-bleaching will cause the CPs to be ‘artificially’ imbalanced for *both LL and HL cases*. However, as suggested by Supplementary Figure 1 in the SI, photo-bleaching can only account for ~5% of CP imbalance. Thus, imbalances in the pump and probe laser spectrum seems to be the dominant cause for the CP imbalance seen in Fig. 3c. This is also likely because the CPs in *both*, HL and LL, 2D spectra are imbalanced such that CP21 is stronger than CP12, whereas photo-bleaching distorts the 2D spectrum in the opposite way (CP12 becomes slightly stronger relative to CP21).

Supplementary Note 11 – 2D peak shape differences in F-2DES versus conventional 2DES, t_2 = 0 fs cross-peaks

The F-2DES approach offers a complementary method to conventional 2DES spectroscopy for characterizing electronic structure and coupling. The approach has been applied in a small number of studies and there has not yet been much theoretical work to describe fluorescence-detected 2D signals. In conventional 2D spectroscopy, cancellations[22] between (negative) doubly-excited state signal and (positive) ground and excited state signals, can sometimes result in the absence of ground state bleaching induced cross-peaks at t_2 = 0 fs (for example, see Fig. 2 of ref.[23], and upper cross-peak cancellation in Fig. 2 of ref.[24]). As described in this section, in fluorescence-detected 2D, the ground state bleaching signal is not cancelled out by excited state absorption, and correlations between B800 and B850 transitions connected through a common ground state are well-highlighted as t_2 = 0 fs positive cross-peaks between B800 and B850 manifolds, previously masked out in conventional 2DES approaches. Improved theoretical models for LH2 proteins, both detergent-isolated and *in vivo*, will further contribute to the understanding of these t_2 = 0 fs ground state associated cross-peaks, and the nature of initial B800-B850 correlations highlighted by them.

In early pump-probe studies on detergent-isolated LH2 complexes, Sundström and co-workers reported the presence of a negative growing excited state absorption (ESA) signal that grows from t_2 = 0 fs as the energy is transferred from B800 to B850 band in multiple species of purple bacteria (Figs. 2, 3 of ref.[25]). Later, spectrally-resolved pump-probe studies on LH2 complexes, such as those by Cogdell and co-workers[19], showed a strong ESA signal at t_2 = 0 fs (Fig. 4 of ref.[19]). 2D studies on similar systems, for example Fig. 2 in ref. [23], *expectedly* showed 2D peak shapes which are *dominated* by negative signal in the cross-peak regions. Hence, expecting a cross-peak at t_2 = 0 fs in these systems is not unusual and is typically attributed[25] to absorption from B850 manifold to doubly-excited states. What is unusual in fluorescence-detected 2D experiments is the presence of distinct *positive* cross-peaks compared to conventional 2D experiments, for example in Fig. 2 of ref.[23].

Keeping the above observations in mind, it is helpful to analyze the sign of the signals expected in fluorescence-detected 2D experiments. Through analysis of Feynman pathways, Marcus and co-workers have shown that ground state bleaching (GSB) and excited state emission (ESE) signals add with a positive sign. However, in fluorescence-detected case there are two

different kinds of ESA pathways, labelled as ESA_1 and ESA_2 in Supplementary Figure 17. ESA_1 is positively signed and creates a population on singly-excited state and corresponds to Q^*_{2b} in Supplementary Figure 2 of ref.[22]. ESA_2 is negatively signed, and creates a doubly-excited state population, and corresponds to Q^*_{8b} in Supplementary Figure 2 of ref. [22].

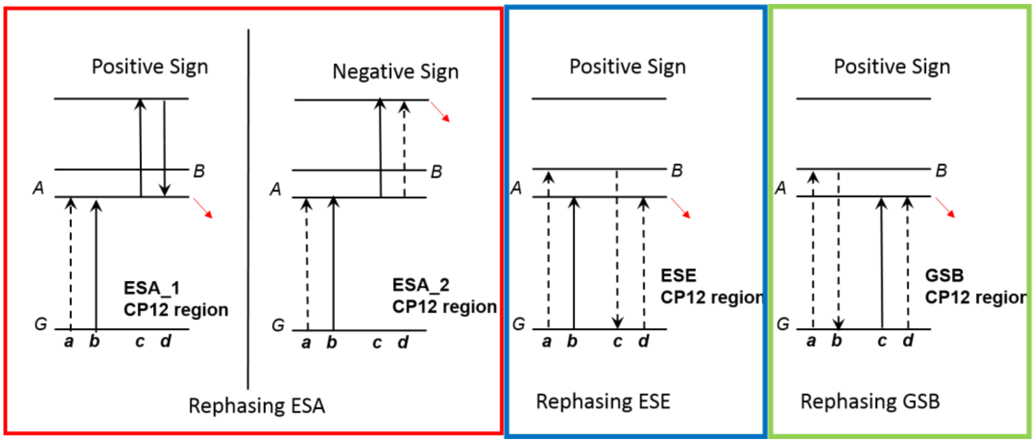

Supplementary Figure 17. Wavemixing diagrams showing ESA (red box), ESE (blue box) and GSB (green box) contributions in fluorescence-detected 2D experiment[22] in the lower CP 'CP12' region. There are two types of ESA contributions, the one shown on the left side in the red box is equivalent to a conventional 2D experiment[7] and creates a population on a singly-excited electronic state after 4 interactions. The ESA contribution shown on the right is an *additional* pathway in fluorescence-detected 2D and creates a population on the doubly-excited state after 4 interactions. The fluorescent population after 4 interactions is denoted as a red arrow. Solid (dashed) arrows can be thought to carry a negative (positive) sign, such that the sign of GSB and ESE becomes the same as ESA_1. This is in contrast to conventional 2D where, GSB and ESE have an opposite sign relative to ESA_1. Thus, ESA_1 signal will add to the ESE and GSB contributions instead of cancelling them. The relative sign of ESA_2 is opposite to that of all other contributions. However, the strength of the ESA_2 contribution depends on the quantum yield of internal conversion of a highly excited *BChl a* monomer, resulting after exciton-exciton annihilation[26-28], to the lowest excited electronic state of *BChl a*. The quantum yield of the internal conversion process dictates the fluorescence quantum yield, and therefore the strength of the ESA_2 pathway. Therefore, the total t_2 = 0 fs contribution on the cross-peaks will have a GSB character, plus some positive mixture of ESA contributions arising from the difference in fluorescence quantum yields of ESA_1 – ESA_2 pathways.

Based on the analysis of wave-mixing pathways outlined in Supplementary Figure 17 (and accompanying caption), the ESA_1 contributions which is known to give rise to negative 2D peak shapes at t_2 = 0 fs is now apriori expected to appear as *positive*, and *add* to the ESE and GSB contributions. The ESA_2 contribution will still be negative, such that the overall character of the t_2 = 0 fs cross-peaks will be GSB plus an admixture of (ESA_1 – ESA_2) pathways, the difference

depending on their relative fluorescence quantum yields, as described in the caption of Supplementary Figure 17. Hence, keeping aside any experimental issues, strong peak shape differences between both 2D approaches are expected, as was also pointed out by Marcus and co-workers[22]. Based on above differences, Marcus and co-workers have simulated a conventional 2D and a fluorescence-detected 2D spectrum for a dimer and fit it to experimental measurements. They report distinct cross-peak in F-2DES which is apparently absent conventional 2DES because of cancellations between negative ESA and positive ESE and GSB signals in conventional 2DES as discussed above.

Very recently, while this paper was in revision, Pullerits and co-workers from Lund University, Sweden, have reported[29] t_2 = 0 fs fluorescence-detected 2D spectrum from detergent-isolated LH2 complexes, and report distinct positive CPs similar to those reported in our *in vivo* measurements. They consider different hypotheses to explain why the t_2 = 0 fs CPs are well-resolved, as opposed to earlier conventional 2D measurements. In agreement with the work of Marcus and our argument above, they also conclude that the cross-peaks as due to the intrinsic difference between fluorescence-detected and conventional 2D approaches. Assuming near unity quantum yield of internal conversion in highly excited *BChl a* electronic states after exciton-exciton annihilation, they propose near perfect cancellation between the two types of ESA pathways, that is, ESA_1 – ESA_2 ~ 0, such that the t_2 = 0 fs cross-peaks are dominantly GSB in character. Recently, Tokmakoff and co-workers[30] have also reported similar differences in the fluorescence encoded 2DIR experiments (compare Fig. 5b,c in ref.[30]).

In the conventional 2DES measurements, t_2 = 0 fs positive GSB cross-peaks between B800-B850 manifolds were masked out by negative ESA signals. Such making effects due to strong ESA signals are also known in other photosynthetic proteins, such as the FMO antenna (for example, some of the early waiting time GSB-like cross-peaks are only revealed through conventional 2DES experiments with a polarization scheme that suppresses strong positive and negative diagonal features in Fig. 2 of ref. [31]). However, as indicated in our data and in the recent report by Pullerits and co-workers[29], there are, in fact, strong cross-peaks at t_2 = 0 fs predominantly due to GSB signals arising out of a common ground state. This opens up new questions about the interpretation of GSB cross-peaks and the common ground state between B800-B850 rings. It should be noted that t_2 ~ 0 fs bleach signals in the B850 band after a narrow band pump excitation

in the B800 band has been observed by Hess et al. (Fig. 7B of ref. [32]), Moulisová et al. (Fig. 3 of ref. [19]), and Niedzwiedzski et al. (Figs. 7, 8 of ref. [33]) although with ~100-200 fs time resolution of the excitation pulse. Thus, t_2 = 0 fs GSB cross-peaks between B800-B850 bands may not be completely unexpected in SF-2DES.

The best atomistic calculations of 2D spectra of LH2 from *Rs. molishianum*, which are based on previous 2D measurements, show a lack of t_2 = 0 fs cross-peaks (for example, see Fig. 8 of ref. [34]). Future work will compare the above simulations of conventional 2DES spectra from Jansen and co-workers, with fluorescence-detected 2D simulations of LH2 in the context of *R. Palustris*. Simulations of the effect of growth conditions on the excitonic structure in the B850/B850* manifolds will also be a subject of future investigations. The B800 to B850 energy transfer is known [35] to be as fast as ~300 fs due to mixing between the higher lying B850* exciton manifold with the B800 manifold. In particular, reduction in the number of B850-like sites and conversion of some of them into B810-like sites under low-light growth conditions must affect the higher lying B850* manifold, and its mixing with the B800 manifold. Further, it is also already known that the ~200 fs B850 intra-band relaxation[35] also slows down to ~2 ps for low-light grown *R. palustris*[19]. Moulisova et al.[19] also estimate the reduction in coupling between closest B850-like sites in a low-light B850 ring to be down to ~50 cm^{-1}, a reduction of ~6x compared to the unperturbed B850 ring in a high light grown bacteria. The mixing between the B850 exciton manifold and the ~850 nm optically dark state[23] in the B800 manifold is also expected to change upon reduction in the number of B850-like sites.

Despite substantial changes in the B850 excitonic structure, and expected changes in the B800-B850 mixing, t_2 = 0 fs GSB like cross-peaks between B800-B850 manifolds are still present in the SF-2DES measurements from low-light cells. This may also be expected based on the spectrally-resolved pump-probe measurements in Fig. 3B of ref. [19]. Note that the LL cells in the present manuscript are closer to the LL1 cells in Moulisova et al.[19] where the B810-like band is ~ 1/4x weaker than the B850-like band. Hence, any possible intra-B850 ring cross-peaks between B810-B850 manifolds and the B810 diagonal peak is expected to be very weak. Our room temperature measurements do not resolve any cross-peaks or diagonal peaks associated with the B810 band, such that the majority of cross-peak contribution is expected to come from the inter-ring B800-B850 interactions. The connection between the t_2 = 0 fs GSB-like cross-peaks, and how

much of a B800-B850 correlation is implied by their presence even for low-light cells, are open questions to be addressed.

Supplementary Note 12 – Exciton Model for Estimating Couplings and Transition Dipole Strength from 2D peaks

We derive an analytic expression to show that growth conditions affect Coulomb coupling and transition dipole strengths, which in turn affect the 2D peak amplitudes seen in the F-2DES measurements. We will do so by oversimplifying the LH2 complex as a dimer, by assuming the B800 and B850 as 'super-molecules' interacting through Coulomb coupling. Note that the oversimplification also neglects the doubly excited manifold of monomeric *BChl a* molecules, which has been shown to be necessary to include[36] in order to correctly simulate the experimentally measured excited-state absorption signals. Because of different excitonic structure[37], the HL and LL type LH2 complexes can be considered as two distinct kinds of dimers. The purpose of this oversimplified dimer model is simply to show how microscopic parameters such as site energy Δ, the hypothesized growth parameter G on which the Coulomb coupling J depends (as described later), are reflected in the various contributions to the 2D peak intensities. Using this dimer model, we will argue that –

i. Presence of CPs in the reported 2D measurements shows that B800 and B850 excitonic manifolds are coupled through a common ground state.
ii. Spatially-varying constitution of HL and LL types of cells cause spatially-varying Coulomb couplings and exciton transition dipole strengths, which in turn map onto spatially-varying 2D peak amplitudes.

To the best of authors' knowledge no 2D experiments and simulations probing a *manifold* of excitonic states, such as the B800 and B850 LH2 bands, have reported an analysis of coupling strength directly based on the strength of experimentally measured 2D cross-peaks. Condensed phase 2D measurements at room temperature have broad peak shapes due to electronic dephasing caused by the protein bath and the molecular vibrational degrees of freedom, and ensemble averaging over an inhomogeneous distribution of excited state energy gaps. Thus, without a protein structure based microscopic Hamiltonian, the experimentally measured 2D CPs will only represent an 'effective' coupling strength between the B850 and B800 manifolds. For each LH2 ring in the ensemble, there is a manifold of 9 B800 like and 18 B850 like excitonic states, with certain

excitonic states having a mixed B800-B850 character[34]. Here we will represent these *excitonic* manifolds as having 'effective' absorption strengths, $|\vec{\mu}_{eff}^{B850}|^2$ and $|\vec{\mu}_{eff}^{B800}|^2$. This simplification will be necessary in order for us to make a tractable estimate of how the Coulomb coupling between any two *BChl a* pairs between the B800 and B850 rings manifests itself in the 2D CPs. In doing so, the effect of relative orientations of constituent *BChl a* within each band, and the symmetric geometry of the rings, is completely neglected. An ab-initio calculation[34] starting from the LH2 protein crystal structure based on a microscopic Hamiltonian can include all the above effects. However, the latest of such calculations for LH2 complex[34] or any other photosynthetic proteins have not tracked down all the eigenstate combinations (and therefore contributions in the site basis from individual *BChl a* sites) which result in a 2D cross-peak. Although daunting, in principle this could be done by counting all the sum-over-all-states non-linear response functions which give rise to a 2D cross-peak. *In vivo* interactions between adjacent LH2s will further complicate things and have not been considered so far in such calculations[34].

We make the above 'simplifying' assumptions and represent the LH2 complex effectively as a dimer. We neglect the ~810 nm which develops for LL cells (Fig. 1D of ref.[19]) . We also do not consider intramolecular vibrations and therefore neglect any possible vibrational-electronic mixing effects[21]. Supplementary Figure 18 (left panel) shows the energy levels of the dimer along with the site basis functions. Two 'super-molecules' *X* and *Y*, representing B800 and B850 manifolds, with the respective transition dipoles $\vec{\mu}_X = \langle 0_X|\vec{\mu}|X\rangle$ and $\vec{\mu}_Y = \langle 0_Y|\vec{\mu}|Y\rangle$, are coupled through Coulomb interaction with interaction energy $J(\theta_{XY},\theta_r,G) = \frac{\sqrt{G}.|\mu_Y|^2}{r^3}\left(\cos\theta_{XY} - 3\cos\theta_r\cos\left(\theta_{XY}-\theta_r\right)\right)$, assuming $|\vec{\mu}_X| = \sqrt{G}.|\vec{\mu}_Y|$. Here G is a growth condition dependent factor which controls the relative strength of B800 and B850 excitonic bands which changes due to changing number of B850-like sites in individual LH2 rings depending on light-stress[37]. θ_{12} and θ_r are defined in Supplementary Figure 18 (right panel). Based on the limited number of studies on *R. palustris* regarding the nature of growth-dependent change in *BChl a* site energies on the B850 LH2 rings[37-39], it is inconclusive whether the there is only a reduction in the total number of B850-like sites, or is it also accompanied by perturbations in the distance or relative orientations of remaining B850-like sites in the LL B850 rings. Here we assume only the former changes upon light-stress, while the relative orientations and distances of the remaining

B850-like sites does not get perturbed under LL conditions. This amounts to having fixed transition dipole angles, and directions between LL and HL cases, such that only the parameter G affects the Coulomb coupling *J*. The excitonic energy gap resulting from the energy levels in Supplementary Figure 18 will be[40] $\Delta_{EX} = \sqrt{4J^2 + \Delta^2}$. The resulting excitons, $|B800\rangle$ and $|B850\rangle$ will be[40] $|B800\rangle = \sin\theta_d |X\rangle|0_Y\rangle + \cos\theta_d |0_X\rangle|Y\rangle$ and $|B850\rangle = \cos\theta_d |X\rangle|0_Y\rangle - \sin\theta_d |0_X\rangle|Y\rangle$.

The respective transition dipoles, $\vec{\mu}_{eff}^{B800}$ and $\vec{\mu}_{eff}^{B850}$ will be[40], $\vec{\mu}_{eff}^{B800} = \sin\theta_d \vec{\mu}_X + \cos\theta_d \vec{\mu}_Y$ and $\vec{\mu}_{eff}^{B850} = \cos\theta_d \vec{\mu}_X - \sin\theta_d \vec{\mu}_Y$. Here, θ_d is the diabatic mixing angle and is defined as $2\theta_d = \tan^{-1}(2J/\Delta)$.

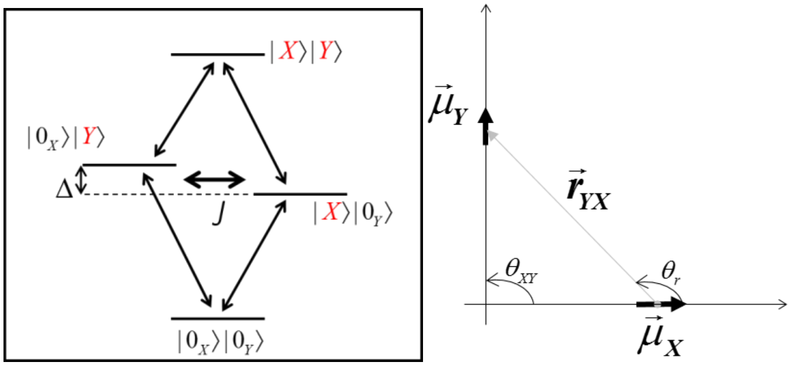

Supplementary Figure 18. (Left) Site energy levels for a purely electronic dimer with molecules '*X*' and '*Y*' coupled through Coulomb interactions between their transition dipoles $\vec{\mu}_X$ and $\vec{\mu}_Y$. The resulting interaction energy is *J*, and depends on the angles θ_{12} and θ_r between the transition dipoles. We will assume co-planar transition dipoles such that only two angles are necessary to describe their interaction energy. Δ is the site energy difference between the molecules. Choice of angles θ_{12} and θ_r dictates whether a dimer behaves like an H or a J aggregate. For simplicity, for the model presented here, we consider θ_{12} = 90° and θ_r = 135° such that the resulting Coulomb coupling is positive, and the site transition dipoles are perpendicular.

For perpendicular site transition dipoles as described in Supplementary Figure 18, the exciton absorption strengths are given by $|\vec{\mu}_{eff}^{B850}|^2 = G\cos^2\theta_d + \sin^2\theta_d$, and $|\vec{\mu}_{eff}^{B800}|^2 = \cos^2\theta_d + G\sin^2\theta_d$. Based on the equilibrium linear absorption spectra in Supplementary Figure 15C, $|\vec{\mu}_{eff}^{B850}|^2 / |\vec{\mu}_{eff}^{B800}|^2 \sim 0.9$ for LL cells and $|\vec{\mu}_{eff}^{B850}|^2 / |\vec{\mu}_{eff}^{B800}|^2 \sim 1.6$ for HL cells, calculated at the peak locations of the B800 and B850 bands in the linear absorption spectrum. Note that, the absorption strengths depend on Coulomb coupling *J* and site energy Δ through the diabatic mixing angle θ_d, as well as on the light-stress dependent factor *G*. From the linear absorption spectrum,

the ratio of absorption strengths and the excitonic energy gap, partly constrain the parameters J, Δ, and G. To calculate these parameters, J can be estimated from the protein structure[34], and Δ and G can be inferred. However, given a linear absorption spectrum with two bands, it is not possible to conclude apriori whether the two bands represent a coupled system, or two independent species. In contrast, a 2D spectrum of a coupled system will show cross-peaks versus no cross-peaks for independent species. In order to calculate the relative strengths of the 2D peaks, the excitonic states $|B800\rangle$ and $|B850\rangle$ derived above, can be plugged into the nonlinear response functions corresponding to the 2D peaks (DP_B800, DP_B850, CP12, CP21).

From Supplementary Figure 19 (A-D) below, shows all the non-oscillatory contributions at DP and CP 2D locations contributing at t_2 = 0 fs in an absorptive 2D spectrum. Each pathways represents the time-evolution of a third order time-dependent density matrix element. The location on a 2D map depends on the 'color' of the first and last interactions.

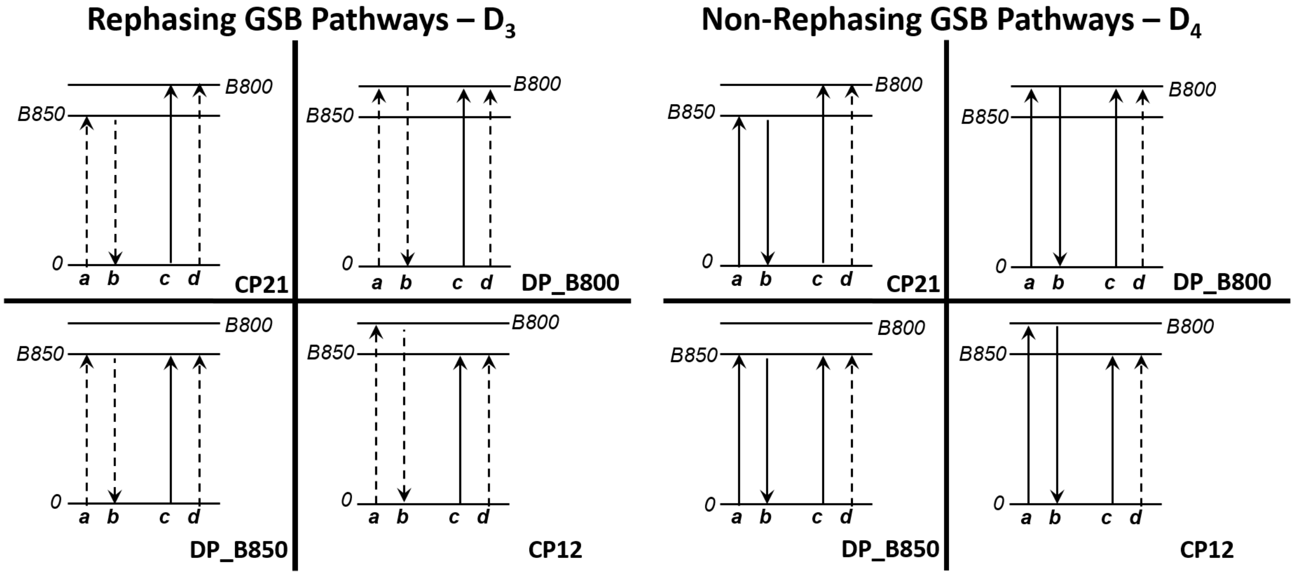

Supplementary Figure 19. (A) Rephasing and Non-rephasing GSB pathways which contribute to the diagonal (DP) and cross-peak (CP) regions in a t_2 = 0 fs absorptive 2D spectrum of the dimer in Supplementary Figure 18. All diagrams have a positive sign and lead to a non-oscillatory signal along the waiting time (time interval between second and third interaction). The diagram notation D_3 and D_4 is adopted from ref.[7].

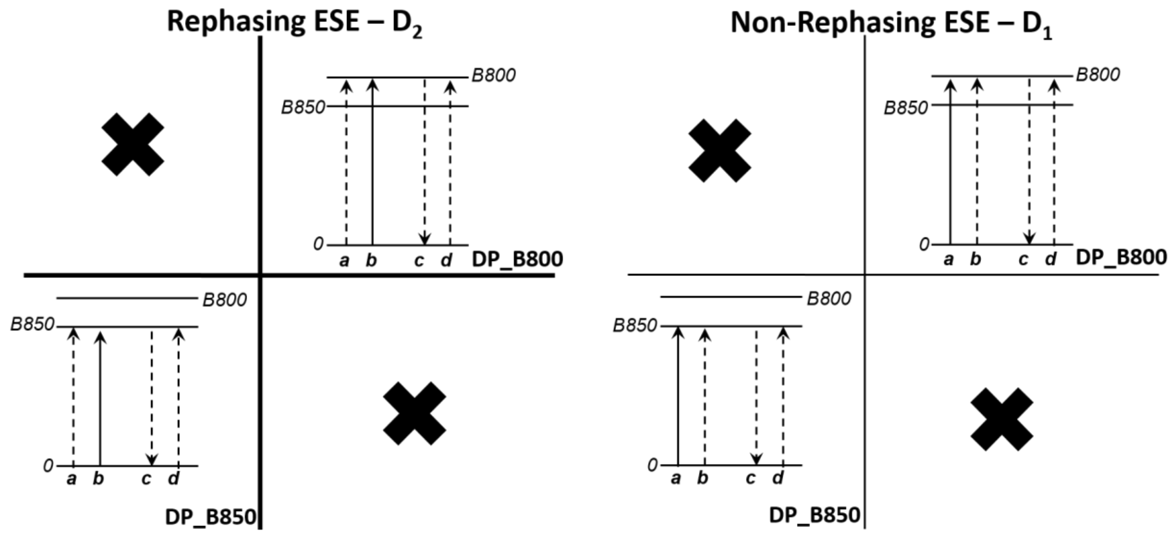

Supplementary Figure 19. (B) Rephasing and Non-rephasing ESE pathways which contribute to the diagonal (DP) and cross-peak (CP) regions in a $t_2 = 0$ fs absorptive 2D spectrum of the dimer in Supplementary Figure 18. All diagrams have a positive sign and lead to a non-oscillatory signal along the waiting time (time interval between second and third interaction). Note that no $t_2 = 0$ fs non-oscillatory ESE pathways contribute to the CP regions. The diagram notation D_2 and D_1 is adopted from ref.[7].

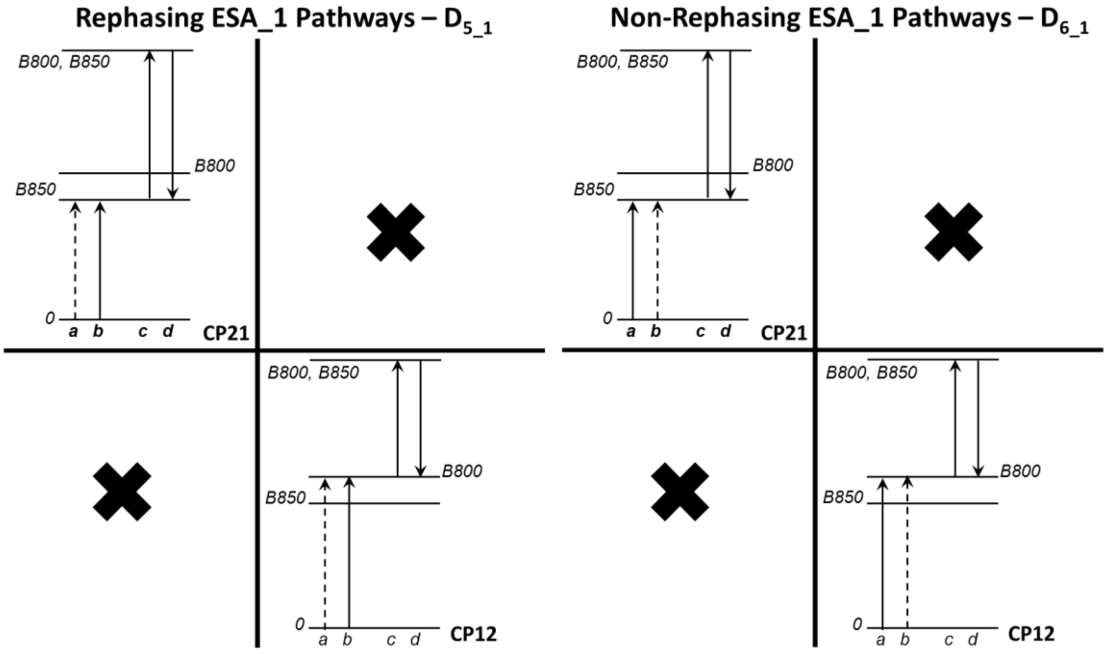

Supplementary Figure 19. (C) Rephasing and Non-rephasing ESA_1 pathways which contribute to the diagonal (DP) and cross-peak (CP) regions in a $t_2 = 0$ fs absorptive 2D spectrum of the dimer in Supplementary Figure 18. All diagrams have a positive sign and lead to a non-oscillatory signal along the waiting time (time interval between second and third interaction). Note that no $t_2 = 0$ fs

non-oscillatory ESA_1 pathways contribute to the DP regions. The diagram notation D_5 and D_6 is adopted from ref.[7], whereas "_1" in the subscript represents the ESA_1 type of F-2DES ESA pathway, as described in SI section "2D peak shape differences in F-2DES versus conventional 2DES, t_2 = 0 fs cross-peaks."

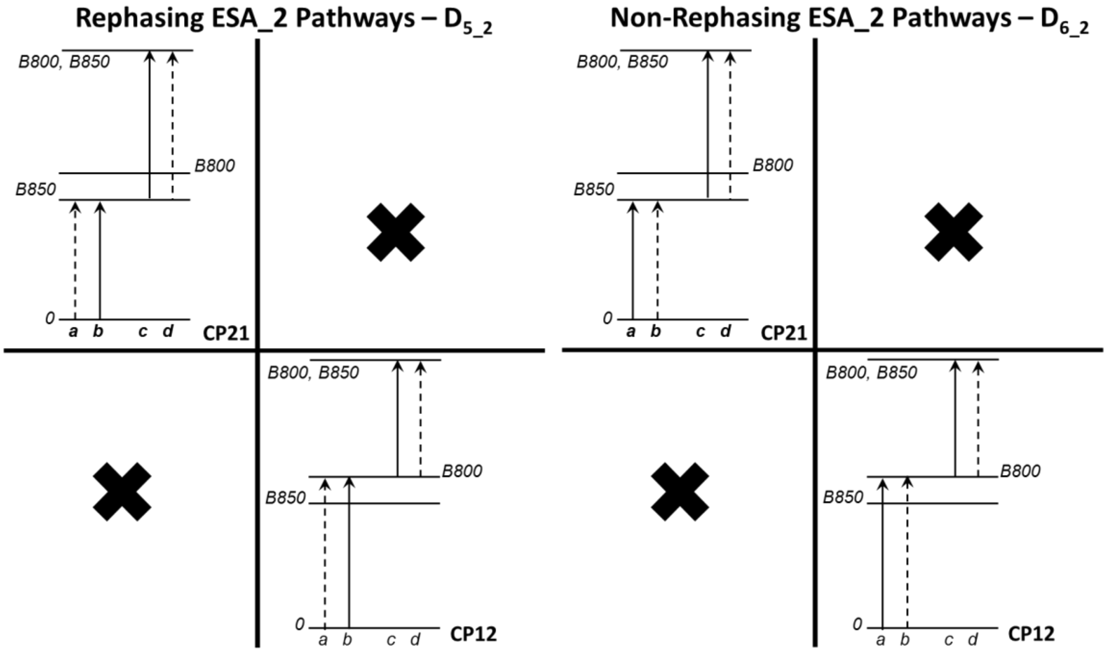

Supplementary Figure 19. (D) Rephasing and Non-rephasing ESA_2 pathways which contribute to the diagonal (DP) and cross-peak (CP) regions in a t_2 = 0 fs absorptive 2D spectrum of the dimer in Supplementary Figure 18. Compared to the diagrams in Supplementary Figures19 (A-C), all ESA_2 diagrams have a negative sign. As described in SI section "2D peak shape differences in F-2DES versus conventional 2DES, t_2 = 0 fs cross-peaks", the signal strength of ESA_2 pathways depends on the quantum yield of internal conversion with a *BChl a* monomer in the LH2 ring. Similar to the modeling by Lott et al.[22], relative to the signal strength of ESA_1 pathway, the strength of ESA_2 pathway will be represented by a parameter Γ. Note that no t_2 = 0 fs non-oscillatory ESA_2 pathways contribute to the DP regions. The diagram notation D_5 and D_6 is adopted from ref.[7], whereas "_2" in the subscript represents the ESA_2 type of F-2DES ESA pathway.

From Supplementary Figures19 (A-D), the 2D peak amplitudes will be given by –

$$DP\_B800 \sim D_3 + D_4 + D_2 + D_1$$
$$DP\_B850 \sim D_3 + D_4 + D_2 + D_1$$
$$CP12 \sim D_3 + D_4 + (D_{5\_1} + D_{6\_1}) - \Gamma(D_{5\_2} + D_{6\_2})$$
$$CP21 \sim D_3 + D_4 + (D_{5\_1} + D_{6\_1}) - \Gamma(D_{5\_2} + D_{6\_2})$$

The GSB pathways (D_3 and D_4) contribute to all peaks, ESE pathways (D_2 and D_1) contribute to only diagonal peaks, and ESA pathways (D_5 and D_6 corresponding to ESA_1 and ESA_2)

contribute to only cross-peaks. At t_2 = 0 fs the rephasing and non-rephasing pathways are balanced and contribute with equal strengths. For an ideal dimer considered above the GSB, ESE and ESA pathways will also be balanced at t_2 = 0 fs, when no population relaxation has happened. For the analysis, we assuming the same laser spectrum for all pulses. Each interaction in a given diagram depends on the product of transition dipole vector with the electric field vector, such as $\vec{\mu}_{0,B800}.\hat{\varepsilon}$ [7]. This product could be simplified as $|\vec{\mu}_{0,B800}|(\hat{\mu}_{0,B800}.\hat{\varepsilon})$, such that the part in the parenthesis contributes to the orientational average and the dipole magnitude contribute to the strength of a given pathway. Based on the dipole products the strength of each 2D peak will be –

$$DP\_B800 \sim 4|\vec{\mu}_{0,B800}||\vec{\mu}_{B800,0}||\vec{\mu}_{B800,0}||\vec{\mu}_{0,B800}| = 4|\vec{\mu}_{B800,0}|^4 = 4\left(\cos^2\theta_d + G\sin^2\theta_d\right)^2$$

$$DP\_B850 \sim 4|\vec{\mu}_{0,B850}||\vec{\mu}_{B850,0}||\vec{\mu}_{B850,0}||\vec{\mu}_{0,B850}| = 4|\vec{\mu}_{B850,0}|^4 = 4\left(G\cos^2\theta_d + \sin^2\theta_d\right)^2$$

$$\begin{aligned} CP12 \sim (4-2\Gamma)|\vec{\mu}_{0,B800}||\vec{\mu}_{B800,0}||\vec{\mu}_{B850,0}||\vec{\mu}_{0,B850}| &= (4-2\Gamma)|\vec{\mu}_{B850,0}|^2|\vec{\mu}_{B800,0}|^2 \\ &= (4-2\Gamma)\left(\cos^2\theta_d + G\sin^2\theta_d\right).\left(G\cos^2\theta_d + \sin^2\theta_d\right) \end{aligned}$$

$$\begin{aligned} CP21 \sim (4-2\Gamma)|\vec{\mu}_{0,B850}||\vec{\mu}_{B850,0}||\vec{\mu}_{B800,0}||\vec{\mu}_{0,B800}| &= (4-2\Gamma)|\vec{\mu}_{B850,0}|^2|\vec{\mu}_{B800,0}|^2 \\ &= (4-2\Gamma)\left(\cos^2\theta_d + G\sin^2\theta_d\right).\left(G\cos^2\theta_d + \sin^2\theta_d\right) \end{aligned}$$

As seen from the above equations, strength of each t_2 = 0 fs 2D peak is proportional to the exciton transition dipole strength, which in turn directly depends on the microscopic parameters *G*, *J* and Δ. When the Coulomb coupling *J* changes due to changes in *G*, this is directly reflected in the 2D peak amplitudes. *Note that the dimer model in Supplementary Figure 18 already assumes a common ground state for molecules X and Y, implying that the molecules are necessarily correlated through Coulomb coupling in this case.* This constraint allows CP pathways through a *common* ground state such as GSB CP pathways in Supplementary Figure 19, panel A. Hence, the presence of CPs reflect correlations between the two molecules. If the molecules were not coupled, there will be no common ground state and therefore no such correlations, that is, no CPs.

To summarize, the presence of CPs reflects that the molecules are coupled, and the amplitude of CPs reflects the magnitude of coupling. When the sample is spatially heterogeneous (such as in the reported measurements), the presence of CPs already reflects that B800 and B850 manifolds are coupled, and the spatial variations in CP and DP amplitudes directly reflect the spatial variations in parameters Δ, G and J.

The above equations for 2D peak amplitudes at $t_2 = 0$ fs can be further simplified assuming a normalized B800 peak (as in Fig. 2b in the manuscript for LL cells), or a normalized B850 peak (as in Fig. 2c in the manuscript for HL cells), such that –

$$DP\_B800 \equiv 1$$
$$DP\_B850 \equiv \left(\frac{G + \tan^2 \theta_d}{1 + G \tan^2 \theta_d} \right)^2$$
$$CP12 \equiv (1 - \Gamma / 2) \left(\frac{G + \tan^2 \theta_d}{1 + G \tan^2 \theta_d} \right)$$
$$CP21 \equiv (1 - \Gamma / 2) \left(\frac{G + \tan^2 \theta_d}{1 + G \tan^2 \theta_d} \right)$$

OR

$$DP\_B800 \equiv \left(\frac{1 + G \tan^2 \theta_d}{G + \tan^2 \theta_d} \right)^2$$
$$DP\_B850 \equiv 1$$
$$CP12 \equiv (1 - \Gamma / 2) \left(\frac{1 + G \tan^2 \theta_d}{G + \tan^2 \theta_d} \right)$$
$$CP21 \equiv (1 - \Gamma / 2) \left(\frac{1 + G \tan^2 \theta_d}{G + \tan^2 \theta_d} \right)$$

For a simple dimer system, the above equations show how the microscopic parameters *G*, *J* and Δ, map onto the 2D peak intensities. Note that the above equations are not independent, and predict that both 2D CPs should be of equal strengths, with CP strengths being related to the strength of the ‘B800’ or ‘B850’ diagonal peaks. The absolute strengths of the CPs also depend on the relative strengths of the ESA_1 and ESA_2 pathways, reflected by the parameter Γ. For more complex systems, deviations from the predicted ratios are already expected, as described in the introduction to the model, since only a purely electronic dimer model, starting with two coupled two-level systems, has been described here. In case of LH2, neglecting the underlying ~810 nm band for LL cells implies that such deviations would be even bigger for the LL case. Imbalance between 2D peak amplitudes are also known to be caused[21] when vibrational modes are included in the model, or red-shift of ESE or ESA cross-sections along the detection axis. Vertical distortions in the 2D spectrum, as seen in Supplementary Figure 1, accounts for ~5% or less of the imbalance between relative peak amplitudes. The above analysis also assumes a flat laser spectrum covering both B800 and B850 bands, thus neglecting the role of the laser spectrum in determining 2D peak amplitudes and positions as discussed in Supplementary Figure 15.

The above equations for 2D amplitudes expected from a purely electronic dimer, and the experimentally derived amplitudes below *constrain the relation between diabatic mixing angle* θ_d *and the growth parameter G.* In order to determine θ_d and G independently, either the site energy Δ, or the Coulomb coupling J is required from a quantum mechanical electronic-structure calculation for the dimer.

Similar to the mapping of microscopic parameters G, J and Δ onto the 2D peaks, as described above for a purely electronic dimer, these parameters will also modulate the 2D peak intensities for a system as complex as the LH2 protein. However, the peak ratios expected from the above equations will no longer hold for a complex molecular system. For the LH2 protein, a protein-structure based calculation, including all the 27 *BChl a* molecules and their doubly-excited states in the Hamiltonian, will be required to make any quantitative predictions.

In conclusion –

1. CPs in the reported 2D measurements show that B800 and B850 excitons are coupled.
2. Growth conditions affect coupling between the excitons and their transition dipole strengths. 2D peak amplitudes reflect those changes.
3. Spatial variations in growth conditions (that is, heterogeneity in HL to LL cells ratio) map onto spatial variations of couplings and transition dipole strengths, and are therefore reflected by spatially-varying 2D peak amplitudes.

In the current 2D study, spatial variations in 2D peak amplitudes are ultimately caused by spatially-varying HL to LL cells ratio. In other systems spatial variations in 2D spectrum could be caused by other factors, such as the presence of grain boundaries in perovskite thin films (ref. 33 of the manuscript), which cause the transient absorption signal to change in sign and magnitude at those locations. In the same way as presented in the current manuscript, resolving such locations on a 2D map can show the presence of additional states or couplings.

Supplementary Note 13 – Modeling of Repetition Rate Dependent Populations

The triplet yield of chlorophylls and its derivative molecules is >60% in solution[41-43]. Intersystem crossing to triplet electronic states is induced by mixing between orbital and spin angular momentum of an electron. This is a well-studied phenomenon in large planar aromatic molecules, and it is further accentuated in chlorophylls due to a heavy central Magnesium atom[44]. The intersystem crossing times in *Bacteriochlorophyll a* (*BChl a*) molecule is ~5-10 ns[13,45]. In the absence of other photo-physical processes, the photophysics for *BChl a* monomers in solution can be well described using Model A (below).

<u>Model A</u>

Supplementary Figure 20 (below, top panel) describes the kinetic scheme for Model A. The intersystem crossing timescale between an excited triplet B850 *BChl a* molecule back to the singlet ground electronic state is known to be ~100μs in solution, and the population relaxation from excited singlet *BChl a* to singlet ground state is ~3 ns in solution. Both timescales have been taken directly from those measured in Ref. [43] in solutions at room temperature. Later, we contrast Model A with the situation expected *in vivo*[46] (in the presence of Carotenoid molecules and surrounding LH2 and LH1-RC complexes).

Based on the Model A kinetic scheme, Supplementary Figure 20 (below, bottom panel, left) shows the expected populations remaining (on nanosecond timescales or longer) in *BChl a* singlet or triplet excited states, *1B850** and *3B850**, respectively, after one laser pulse excites the sample. As expected, the initially created population in *1B850** decays back to the 1B850 ground state, along with a simultaneous rise of long-lived triplet 3B850* states. Supplementary Figure 20 (below, bottom panel, right) considers the additive effect of successive pulses (separated by 83 MHz or ~12 ns), and calculates the steady state population fraction of triplet states to be 100 %. This is expected because the triplet lifetime is much greater than the pulse repetition rate (10^5 ns versus 12 ns). Thus, after ~200 μs, all of the *BChl a* molecules immobilized under the laser spot will be in an excited triplet state.

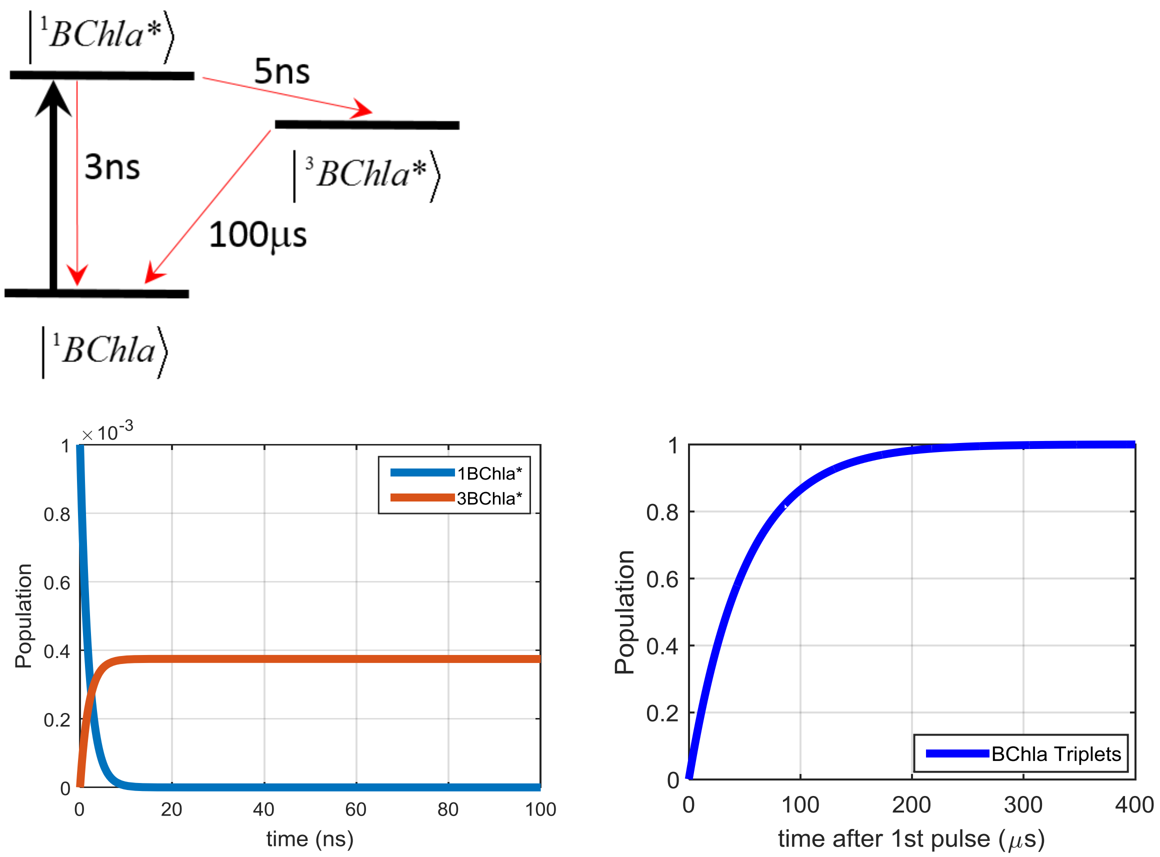

Supplementary Figure 20 (top) – A simplified three energy level scheme to analyze the slow photo-physical processes in a *BChl a* molecule in solution. The time constants have been taken from ref.[43]. (bottom left) Evolution of population after excitation by a single laser pulse. The total ground state population is assumed to be 1, such that excitation probability of 0.1% (as used in the reported experiments) will create 10^{-3} excited state *1B850** population at time zero. The total ground state population is assumed to be 1, such that the excitation probability of 0.1% (as used in the reported experiments) will create 10^{-3} excited state *1B850** population at time zero. (bottom right) Steady state fraction of *BChl a* triplets after a 12 ns (83 MHz) pulse train is incident on an immobilized sample.

Using Models B and C described below, we show that the situation in the reported SF-2DES measurements deviates considerably from that described by Model A, finding that triplet build-up and contributions to the SF-2DES signal are considerably reduced in whole purple bacteria compared to monomeric *BChl a*.

<u>Model B</u>

Model B builds upon Model A and considers a *BChl a* molecule inside detergent-isolated LH2 complexes. For the purpose of describing slow photo-physical processes in the LH2 complex, rapid (~500 fs, 100% efficient)[47] energy transfer from B800 ring to the B850 ring allows us to ignore

the *BChl a* molecules in the B800 ring. The B850 excitonic manifold (comprised of 18 *BChl a* molecules) of the LH2 complex is represented by a single energy level, $^{1}B850$ and $^{1}B850^{*}$, for the ground and excited singlet states, and $^{3}B850^{*}$ for the excited triplet state. *BChl a* molecules in solution versus on the B850 ring are different in two key respects –

1. *BChl a* molecules in the B850 ring are strongly dimeric due to Coulomb coupling. This effect is manifested in increased oscillator strengths causing 3-4x increased radiative rates of *BChl a* fluorescence from LH2 or LH1 complexes. Thus, the triplet quantum yield of a *BChl a* molecule on the B850 ring is estimated to be significantly reduced to ~25%[45].
2. *BChl a* in the B850 ring are in Van der Walls contact with carotenoid molecules which are known[47] to be highly efficient (100% efficient) ^{3}B850* triplet quenchers. Triplet carotenoid molecules are represented by $^{3}Car^{*}$.

Both of the above differences are captured by the parameters of Model B which are taken from the measurements reported by Cogdell, Hashimoto and co-workers[45]. The energy level scheme is shown in Supplementary Figure 21 (below, top left panel). Models that build up from this simplified energy relaxation scheme by partly incorporating multi-chromophoric nature of LH2 complex, and including photo-physical processes such as singlet-triplet and singlet-singlet annihilation, have been analyzed in detail by Kohler and co-workers[13-15] and show quantitative agreement between simulations and experimentally measured fluorescence quenching in LH2 complexes under variable high laser repetition rates and excitation fluences. Pulse fluences used in the current study (64 x 10^{12} photons/cm^2) are more than 5x lower than fluences for which singlet-singlet annihilation has been reported by Pullerits and co-workers[48]. Hence, singlet-singlet annihilation can be neglected. The present model also neglects singlet-triplet and triplet-triplet annihilation processes. Under high triplet and singlet concentrations, both processes start to dominate and the timescales for both processes is expected to be on the order of a few nanoseconds or shorter[13,15]. By neglecting those triplet decay channels, the triplet concentrations predicted by Model 2 should be an overestimate.

Based on the results of Model 2 shown in Supplementary Figure 21, the steady state fraction of population of *BChl a* triplets is minimal owing to efficient quenching by adjacent carotenoid molecules such that in the steady state all the carotenoid molecules in the B850 rings are converted into triplets. For similar repetition rates and fluences as reported here, experimental

measurements and Monte Carlo simulations of Kohler and co-workers have estimated[13] ~ 90% steady state carotenoid triplet in the detergent isolated LH2 rings. Comparing Model A and Model B, and considering the study by Kohler and co-workers[13] some qualitative conclusions can be derived for LH2 rings excited by a high repetition rate laser –

1. Steady state fraction of *BChl a* triplets in LH2 rings is minimal, while nearly all the adjacent carotenoids are converted to triplets.
2. Under high repetition rates, triplet-triplet and singlet triplet annihilation dominate leading to discrepancies between experiments and simulated estimates of fluorescence quenching (as reported in ref. [13]) possibly due to underestimation of annihilation rates.

Both of the above conclusion are markedly different from Model A (of isolated *BChl a* molecules in solution), and are a result of the built-in photo-protection machinery of the LH2 proteins[13].

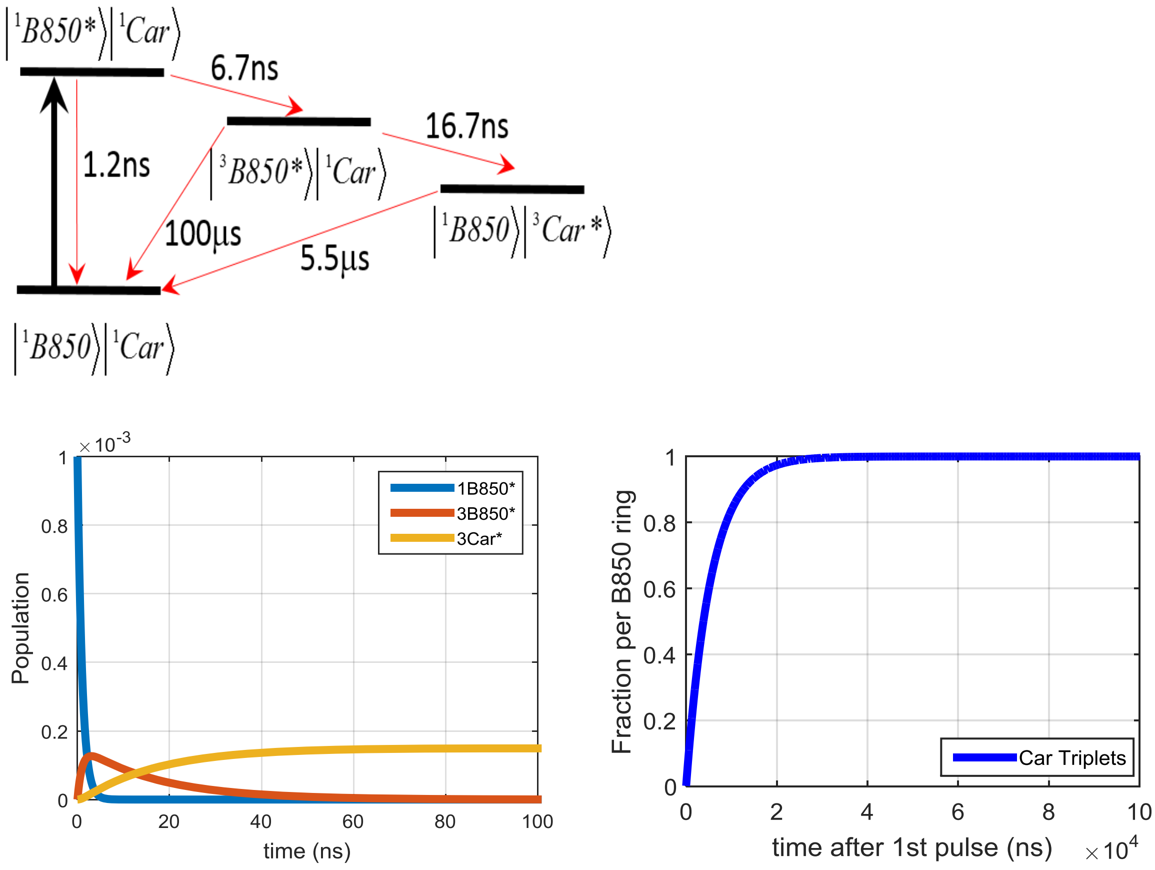

Supplementary Figure 21 (top) – A simplified energy level scheme to analyze the slow photo-physical processes in a *BChl a* molecule in the B850 ring. The time constants have been taken from ref.[45]. (bottom left) Evolution of population after excitation of a *BChl a* molecule on B850

LH2 rings by a single laser pulse. The total ground state population is assumed to be 1, such that the excitation probability of 0.1% (as used in the reported experiments) will create 10^{-3} excited state *1B850** population at time zero. (bottom right) Steady state fraction of Carotenoid triplets after a 12 ns (83 MHz) pulse train is incident on an immobilized sample, and excitation probability per LH2 ring (that is, excitation probability per *BChl a* x 18 *BChl a* molecules per B850 LH2 ring) is considered[13].

Model C

Model C builds upon Model B by considering LH2 complex *in vivo*. In purple bacterial photosynthetic cells, the transfer of excitations from LH2 proteins to the LH1-RC complex is complete within ~10 ps for HL *R. palustris* cells and nearly ~18 ps for LL *R. palustris* cells, as reported by Cerullo, Cogdell and co-workers[20]. They have also measured back energy transfer rates from LH1 to LH2 complexes, and shown that a semi-equilibrium exists between LH1 and LH2 complexes, *which also indicated absence of LH2 complexes without any energy transfer pathways towards LH1*. For Model C, the kinetic rates pertaining to LH2 and LH1 are taken from ref.[20] for estimating the amount of carotenoid triplets on LH2 rings in the presence of fast (forward and backward) population transfer towards LH1 LL and HL *R. palustris* cells.

Repetition rate and fluence dependent photo-physics within isolated LH1-RC complexes has been studied extensively by Kohler and co-workers[14] through quantitative modeling based on a global master equations approach. Earlier work from the same group has studied similar effects on isolated LH2 complexes[13] and LH2 complexes in arrays[15] (but without an LH1-RC 'sink'). Here, we have considered a very simplified approach and neglected the singlet-triplet (and triplet-triplet) quenching dynamics within the LH1-RC complex. Conclusions about the triplet quenching dynamics within the LH1-RC complex from ref.[14] will further add to the picture derived from Model C. We *combine* the simplified LH2 photo-physics (derived from Model B) with the presence of a LH1-RC 'sink' which is equilibrated[20] with an LH2 ring. For fluences and repetition rates used in the present study, Kohler and co-workers estimate that >90% of the RC proteins will be in an oxidized P^+ state (Fig. 9A of ref.[14]). For oxidized RC proteins, the timescale of energy transfer from LH1 to closed RCs (RC+*) is estimated by ref.[14] to be ~0.3 ns. Relaxation of RC+* to the ground state is estimated to be ~0.1 ns from Table 2 of ref.[14] Both pathways are included in Model C. The kinetic scheme for Model C is shown in Supplementary Figure 22 (below).

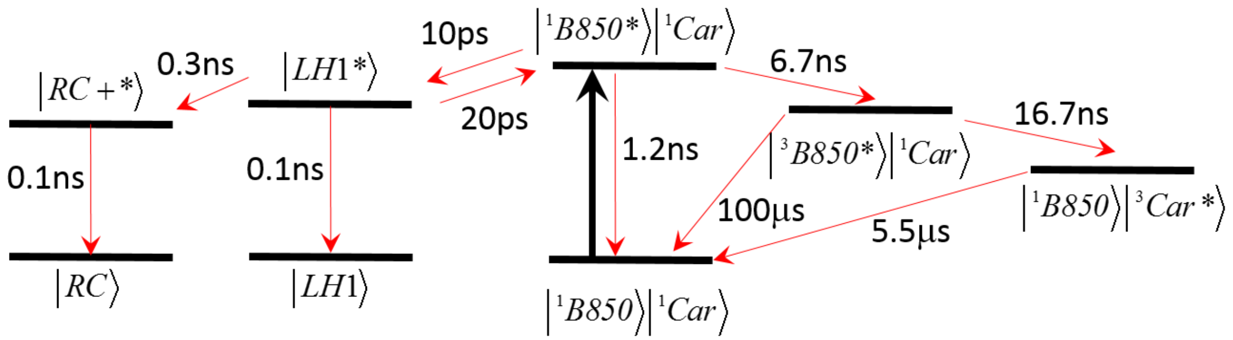

Supplementary Figure 22. Kinetic scheme for Model C based on the findings of refs.[20,45]. The rates constants for LH1 and B850 LH2 rings are for HL *R. palustris* cells[20]. LL *R. palustris* cells also have approximately similar rate constants (see ref.[20] and text below), such that the calculated fraction of carotenoid triplets on LH2 rings stays approximately the same for both kinds of cells.

The kinetic scheme in Supplementary Figure 22 neglects the triplet quenching dynamics within the LH1-RC complex, and represents an excited LH1 and RC complex as an excited or ground state singlet. The overall effect of neglecting the triplet annihilation pathways should be an *overestimation* of the fraction of carotenoids triplets remaining on the B850 rings.

Supplementary Figure 23 (left) below shows that, following an excitation by a laser pulse, population of *BChl a* singlets on B850 LH2 rings and *LH1** singlets decays rapidly to LH1 ground state (within 1 ns). The triplet population of B850 *BChl a* and carotenoids (shown in Supplementary Figure 23 (right)) is reduced by ~25x in the presence of LH1-RC 'sink' (compare Supplementary Figure 21 bottom left panel with Supplementary Figure 23 right panel).

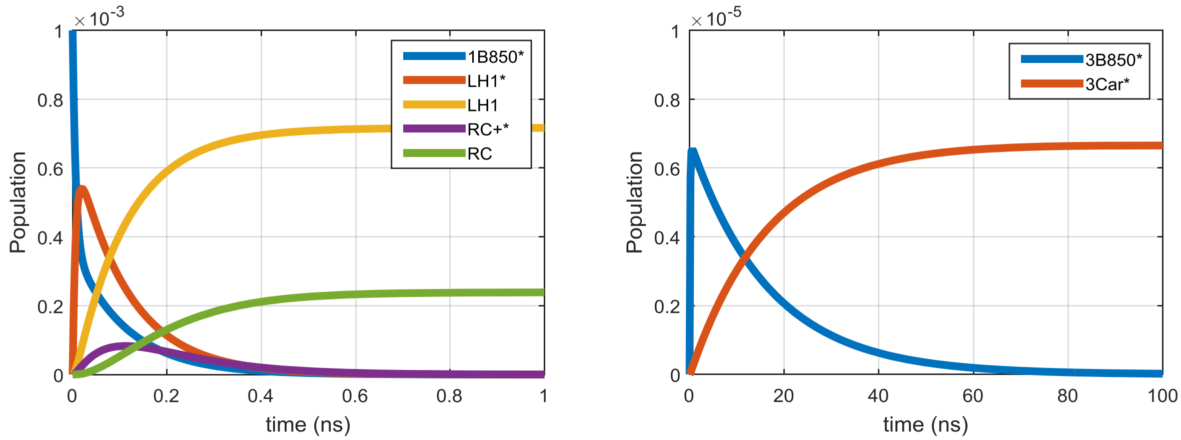

Supplementary Figure 23. Evolution of population after excitation of a *BChl a* molecule on B850 LH2 rings by a single laser pulse. The total ground state population is assumed to be 1, such that

the excitation probability of 0.1% (as used in the reported experiments) will create 10^{-3} excited state *1B850** population at time zero. The figure legend points to the species considered in the kinetic scheme shown in Supplementary Figure 22.

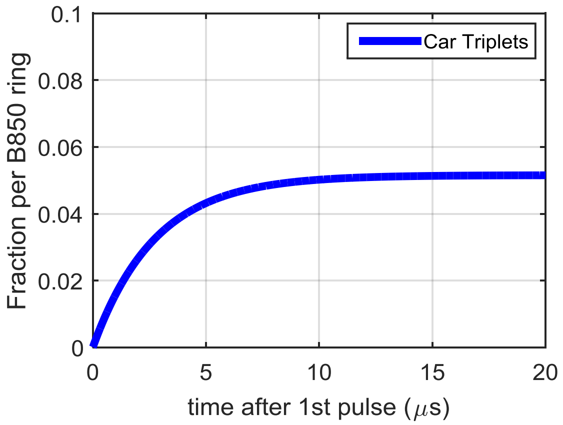

Supplementary Figure 24. Steady state fraction of Carotenoid triplets after a 12 ns (83 MHz) pulse train is incident on an immobilized sample, and excitation probability per LH2 ring (that is, excitation probability per *BChl a* x 18 *BChl a* molecules per B850 LH2 ring[13]) is considered.

From the action of one laser pulse in Supplementary Figure 23, Supplementary Figure 24 calculates the steady state fraction of *BChl a* and carotenoid triplets remaining on a LH2 ring when a laser pulse train of 83 MHz is incident on the sample. As in Supplementary Figure 21, for each laser pulse the excitation probability is considered with respect to each LH2 ring, that is, 18x higher excitation probability, and the additive effect of each pulse gives a steady state fraction of *BChl a* and carotenoid triplets on the LH2 ring immobilized under the laser pulse. *As seen from Supplementary Figure 24, due to the presence of LH1-RC 'sink', the ~100% saturated carotenoid steady state population on LH2 (in Supplementary Figure 21) is now reduced to only ~5%, that is, ~1 carotenoid per LH2 B850 ring.* Note that inclusion of triplet annihilation processes (~10ns[14]) will further reduce the estimated ~5% steady state fraction of carotenoid triplets. Kohler and co-workers note that both, singlet-triplet and triplet-triplet, annihilation process dominate under high repetition rates and give rise to discrepancies between experimentally measured and simulated fluorescence quenching rates (for example, see Fig. 3 of ref. [14] for RC-LH1 complexes, Fig. 8C of ref. [15] for LH2 arrays and Table 2 of ref. [13] for isolated LH2 complexes).

With regard to the dynamics within the LH1-RC complexes, once singlet population is transferred from the B850 complex to LH1, estimations of ref. [14] for approximately the same repetition rates and fluences can be directly applied to the present study. Based on measured and

simulated fluorescence decays from isolated LH1-RC complexes at repetition rates and fluences similar to those in this study, Kohler and co-workers estimate[14] ~40% steady state fraction of all LH1-RC complexes with at least one carotenoid triplet.

Comparing Models A-C and studies by Kohler and co-workers[13-15], we derive the following qualitative conclusions–

i) The scenario of 100 % *BChl a* population converted to triplets under high repetition rates (Model A), only applies to isolated *BChl a* molecules immobilized under the laser.
ii) When *BChl a* molecules are present *in vivo* inside LH2 and LH1 rings, processes[13-15] such as a.) triplet quenching by carotenoid molecules, b.) singlet-triplet annihilation caused by mobile singlets and immobile triplets, c.) triplet-triplet annihilation between adjacent carotenoid molecules, d.) rapid energy transfer towards LH1-RC complexes, will reduce the amount of *BChl a* triplets in LH2 to ~zero, and the amount of carotenoid triplets to <5% per LH2 ring.
iii) A majority of population transfer occurs towards LH1 causing ~40% of LH1 complexes to have one more carotenoid triplets. However, we note that the LH1 band is negligibly probed in the present experiment (the LH1 shoulder is present at 883 nm and there is no laser intensity beyond 870 nm).

The above comparisons indicate that the overall effect of steady state *BChl a* and carotenoid triplet populations will be negligible when probing *LH2* complexes *in vivo* as in the reported measurements. At steady state, most of the LH2 *BChl a* and Car molecules are expected to be present in their ground electronic state. This conclusion based on modeling is consistent with our experiments on flowing samples and our repetition-rate-dependent experiments.

References

(1) Tekavec, P. F.; Lott, G. A.; Marcus, A. H. Fluorescence-detected two-dimensional electronic coherence spectroscopy by acousto-optic phase modulation. *The Journal of Chemical Physics* **2007**, *127*, 214307.

(2) Alber, G. M.; Marshall, A. G. Effect of Sampling Rate on Fourier Transform Spectra: Oversampling is Overrated. *Appl. Spectrosc.* **1990**, *44*, 1111-1116.

(3) Mampallil, D. Some physics inside drying droplets. *Resonance* **2014**, *19*, 123-134.

(4) Cai, Z.; Chattopadhyay, N.; Liu, W. J.; Chan, C.; Pignol, J.-P.; Reilly, R. M. Optimized digital counting colonies of clonogenic assays using ImageJ software and customized macros: Comparison with manual counting. *International Journal of Radiation Biology* **2011**, *87*, 1135-1146.

(5) T., W. Resolution and optical sectioning in the confocal microscope. *Journal of Microscopy* **2011**, *244*, 113-121.

(6) Bartholdi, E.; Ernst, R. R. Fourier spectroscopy and the causality principle. *Journal of Magnetic Resonance (1969)* **1973**, *11*, 9-19.

(7) Jonas, D. M. Two-Dimensional Femtosecond Spectroscopy. *Annual Review of Physical Chemistry* **2003**, *54*, 425-463.

(8) Augulis, R.; Zigmantas, D. Two-dimensional electronic spectroscopy with double modulation lock-in detection: enhancement of sensitivity and noise resistance. *Opt. Express* **2011**, *19*, 13126-13133.

(9) Dostál, J.; Pšenčík, J.; Zigmantas, D. In situ mapping of the energy flow through the entire photosynthetic apparatus. *Nature Chemistry* **2016**, *8*, 705.

(10) Dahlberg, P. D.; Ting, P.-C.; Massey, S. C.; Allodi, M. A.; Martin, E. C.; Hunter, C. N.; Engel, G. S. Mapping the ultrafast flow of harvested solar energy in living photosynthetic cells. *Nature Communications* **2017**, *8*, 988.

(11) Sohail, S. H.; Dahlberg, P. D.; Allodi, M. A.; Massey, S. C.; Ting, P.-C.; Martin, E. C.; Hunter, C. N.; Engel, G. S. Communication: Broad manifold of excitonic states in light-harvesting complex 1 promotes efficient unidirectional energy transfer in vivo. *The Journal of Chemical Physics* **2017**, *147*, 131101.

(12) Zinter, J. P.; Levene, M. J. Maximizing fluorescence collection efficiency in multiphoton microscopy. *Opt. Express* **2011**, *19*, 15348-15362.

(13) Pflock, T. J.; Oellerich, S.; Southall, J.; Cogdell, R. J.; Ullmann, G. M.; Köhler, J. The Electronically Excited States of LH2 Complexes from Rhodopseudomonas acidophila Strain 10050 Studied by Time-Resolved Spectroscopy and Dynamic Monte Carlo Simulations. I. Isolated, Non-Interacting LH2 Complexes. *The Journal of Physical Chemistry B* **2011**, *115*, 8813-8820.

(14) Beyer, S. R.; Müller, L.; Southall, J.; Cogdell, R. J.; Ullmann, G. M.; Köhler, J. The Open, the Closed, and the Empty: Time-Resolved Fluorescence Spectroscopy and Computational Analysis of RC-LH1 Complexes from Rhodopseudomonas palustris. *The Journal of Physical Chemistry B* **2015**, *119*, 1362-1373.

(15) Pflock, T. J.; Oellerich, S.; Krapf, L.; Southall, J.; Cogdell, R. J.; Ullmann, G. M.; Köhler, J. The Electronically Excited States of LH2 Complexes from Rhodopseudomonas acidophila Strain 10050 Studied by Time-Resolved Spectroscopy and Dynamic Monte Carlo Simulations. II. Homo-Arrays Of LH2 Complexes Reconstituted Into Phospholipid Model Membranes. *The Journal of Physical Chemistry B* **2011**, *115*, 8821-8831.

(16) Cho, B.; Tiwari, V.; Jonas, D. M. Simultaneous All-Optical Determination of Molecular Concentration and Extinction Coefficient. *Analytical Chemistry* **2013**, *85*, 5514-5521.

(17) Cho, B.; Tiwari, V.; Hill, R. J.; Peters, W. K.; Courtney, T. L.; Spencer, A. P.; Jonas, D. M. Absolute Measurement of Femtosecond Pump–Probe Signal Strength. *The Journal of Physical Chemistry A* **2013**, *117*, 6332-6345.

(18) Grechko, M.; Zanni, M. T. Quantification of transition dipole strengths using 1D and 2D spectroscopy for the identification of molecular structures via exciton delocalization: Application to α-helices. *The Journal of Chemical Physics* **2012**, *137*, 184202.

(19) Moulisová, V.; Luer, L.; Hoseinkhani, S.; Brotosudarmo, T. H. P.; Collins, A. M.; Lanzani, G.; Blankenship, R. E.; Cogdell, R. J. Low Light Adaptation: Energy Transfer Processes in Different Types of Light Harvesting Complexes from Rhodopseudomonas palustris. *Biophysical Journal* **2009**, *97*, 3019-3028.

(20) Lüer, L.; Moulisová, V.; Henry, S.; Polli, D.; Brotosudarmo, T. H. P.; Hoseinkhani, S.; Brida, D.; Lanzani, G.; Cerullo, G.; Cogdell, R. J. Tracking energy transfer between light harvesting complex 2 and 1 in photosynthetic membranes grown under high and low illumination. *Proceedings of the National Academy of Sciences* **2012**, *109*, 1473-1478.

(21) Tiwari, V.; Peters, W. K.; Jonas, D. M. Electronic resonance with anticorrelated pigment vibrations drives photosynthetic energy transfer outside the adiabatic framework. *Proceedings of the National Academy of Sciences* **2013**, *110*, 1203-1208.

(22) Lott, G. A.; Perdomo-Ortiz, A.; Utterback, J. K.; Widom, J. R.; Aspuru-Guzik, A.; Marcus, A. H. Conformation of self-assembled porphyrin dimers in liposome vesicles by phase-modulation 2D fluorescence spectroscopy. *Proceedings of the National Academy of Sciences* **2011**, *108*, 16521-16526.

(23) Ferretti, M.; Hendrikx, R.; Romero, E.; Southall, J.; Cogdell, R. J.; Novoderezhkin, V. I.; Scholes, G. D.; van Grondelle, R. Dark States in the Light-Harvesting complex 2 Revealed by Two-dimensional Electronic Spectroscopy. *Scientific Reports* **2016**, *6*, 20834.

(24) Schröter, M.; Alcocer, M. J. P.; Cogdell, R. J.; Kühn, O.; Zigmantas, D. Origin of the Two Bands in the B800 Ring and Their Involvement in the Energy Transfer Network of Allochromatium vinosum. *The Journal of Physical Chemistry Letters* **2018**, *9*, 1340-1345.

(25) Hess, S.; Feldchtein, F.; Babin, A.; Nurgaleev, I.; Pullerits, T.; Sergeev, A.; Sundström, V. Femtosecond energy transfer within the LH2 peripheral antenna of the photosynthetic purple bacteria Rhodobacter sphaeroides and Rhodopseudomonas palustris LL. *Chemical Physics Letters* **1993**, *216*, 247-257.

(26) Brüggemann, B.; Herek, J. L.; Sundström, V.; Pullerits, T.; May, V. Microscopic Theory of Exciton Annihilation: Application to the LH2 Antenna System. *The Journal of Physical Chemistry B* **2001**, *105*, 11391-11394.

(27) May, V. Kinetic theory of exciton–exciton annihilation. *The Journal of Chemical Physics* **2014**, *140*, 054103.

(28) T., R.; V., M.; V., S.; O., K. Anharmonic Oscillator Approach to the Exciton-Exciton Annihilation Dynamics in Molecular Aggregates. *Journal of the Chinese Chemical Society* **2000**, *47*, 807-819.

(29) Khadga J. Karki; Junsheng Chen; Atsunori Sakurai; Qi Shi; Alastair T. Gardiner; Oliver Kühn; Richard J. Cogdell; Pullerits, T. Unexpectedly large delocalization of the initial excitation in photosynthetic light harvesting. *arXiv:1804.04840 [physics.chem-ph]* **2018**.

(30) Mastron, J. N.; Tokmakoff, A. Fourier Transform Fluorescence-Encoded Infrared Spectroscopy. *The Journal of Physical Chemistry A* **2018**, *122*, 554-562.

(31) Thyrhaug, E.; Žídek, K.; Dostál, J.; Bína, D.; Zigmantas, D. Exciton Structure and Energy Transfer in the Fenna–Matthews–Olson Complex. *The Journal of Physical Chemistry Letters* **2016**, *7*, 1653-1660.

(32) Hess, S.; Akesson, E.; Cogdell, R. J.; Pullerits, T.; Sundström, V. Energy transfer in spectrally inhomogeneous light-harvesting pigment-protein complexes of purple bacteria. *Biophysical Journal* **1995**, *69*, 2211-2225.

(33) Niedzwiedzki, D. M.; Bina, D.; Picken, N.; Honkanen, S.; Blankenship, R. E.; Holten, D.; Cogdell, R. J. Spectroscopic studies of two spectral variants of light-harvesting complex 2 (LH2) from the

photosynthetic purple sulfur bacterium Allochromatium vinosum. *Biochimica et Biophysica Acta (BBA) - Bioenergetics* **2012**, *1817*, 1576-1587.

(34) van der Vegte, C. P.; Prajapati, J. D.; Kleinekathöfer, U.; Knoester, J.; Jansen, T. L. C. Atomistic Modeling of Two-Dimensional Electronic Spectra and Excited-State Dynamics for a Light Harvesting 2 Complex. *The Journal of Physical Chemistry B* **2015**, *119*, 1302-1313.

(35) Novoderezhkin, V.; Wendling, M.; van Grondelle, R. Intra- and Interband Transfers in the B800−B850 Antenna of Rhodospirillum molischianum: Redfield Theory Modeling of Polarized Pump−Probe Kinetics. *The Journal of Physical Chemistry B* **2003**, *107*, 11534-11548.

(36) Pullerits, T.; Chachisvilis, M.; Sundström, V. Exciton Delocalization Length in the B850 Antenna of Rhodobacter sphaeroides. *The Journal of Physical Chemistry* **1996**, *100*, 10787-10792.

(37) Brotosudarmo, T. H. P.; Kunz, R.; Böhm, P.; Gardiner, A. T.; Moulisová, V.; Cogdell, R. J.; Köhler, J. Single-Molecule Spectroscopy Reveals that Individual Low-Light LH2 Complexes from Rhodopseudomonas palustris 2.1.6. Have a Heterogeneous Polypeptide Composition. *Biophysical Journal* **2009**, *97*, 1491-1500.

(38) Ilioaia, C.; Krüger, T. P. J.; Ilioaia, O.; Robert, B.; van Grondelle, R.; Gall, A. Apoprotein heterogeneity increases spectral disorder and a step-wise modification of the B850 fluorescence peak position. *Biochimica et Biophysica Acta (BBA) - Bioenergetics* **2018**, *1859*, 137-144.

(39) Taniguchi, M.; Henry, S.; Cogdell, R. J.; Lindsey, J. S. Statistical considerations on the formation of circular photosynthetic light-harvesting complexes from Rhodopseudomonas palustris. *Photosynthesis Research* **2014**, *121*, 49-60.

(40) Tiwari, V.; Jonas, D. M. Electronic energy transfer through non-adiabatic vibrational-electronic resonance. II. 1D spectra for a dimer. *The Journal of Chemical Physics* **2018**, *148*, 084308.

(41) Niedzwiedzki, D. M.; Orf, G. S.; Tank, M.; Vogl, K.; Bryant, D. A.; Blankenship, R. E. Photophysical Properties of the Excited States of Bacteriochlorophyll f in Solvents and in Chlorosomes. *The Journal of Physical Chemistry B* **2014**, *118*, 2295-2305.

(42) Musewald, C.; Hartwich, G.; Pöllinger-Dammer, F.; Lossau, H.; Scheer, H.; Michel-Beyerle, M. E. Time-Resolved Spectral Investigation of Bacteriochlorophyll a and Its Transmetalated Derivatives [Zn]-Bacteriochlorophyll a and [Pd]-Bacteriochlorophyll a. *The Journal of Physical Chemistry B* **1998**, *102*, 8336-8342.

(43) Teuchner, K.; Stiel, H.; Leupold, D.; Scherz, A.; Noy, D.; Simonin, I.; Hartwich, G.; Scheer, H. Fluorescence and excited state absorption in modified pigments of bacterial photosynthesis a comparative study of metal-substituted bacteriochlorophylls a. *Journal of Luminescence* **1997**, *72-74*, 612-614.

(44) Cohen-Tannoudji, C.; Diu, B.; Lalöe, F.: *Quantum Mechanics*; Wiley-Interscience: Paris, 1977; Vol. 2.

(45) Kosumi, D.; Horibe, T.; Sugisaki, M.; Cogdell, R. J.; Hashimoto, H. Photoprotection Mechanism of Light-Harvesting Antenna Complex from Purple Bacteria. *The Journal of Physical Chemistry B* **2016**, *120*, 951-956.

(46) Sipka, G.; Maróti, P. Photoprotection in intact cells of photosynthetic bacteria: quenching of bacteriochlorophyll fluorescence by carotenoid triplets. *Photosynthesis Research* **2018**, *136*, 17-30.

(47) Blankenship, R. E.: *Molecular Mechanisms of Photosynthesis*; Blackwell Science Ltd., 2002.

(48) Trinkunas, G.; Herek, J. L.; Polívka, T.; Sundström, V.; Pullerits, T. Exciton Delocalization Probed by Excitation Annihilation in the Light-Harvesting Antenna LH2. *Physical Review Letters* **2001**, *86*, 4167-4170.
